# The increased tendency for anemia in traditional Chinese medicine deficient body constitution is associated with the gut microbiome

**DOI:** 10.3389/fnut.2024.1359644

**Published:** 2024-09-18

**Authors:** Yuanjun Liang, Yang Chen, Yanzhao Lin, Wei Huang, Qinwei Qiu, Chen Sun, Jiamin Yuan, Ning Xu, Xinyan Chen, Fuping Xu, Xiaoxiao Shang, Yusheng Deng, Yanmin Liu, Fei Tan, Chunxiang He, Jiasheng Li, Qinqin Deng, Xiaoxuan Zhang, Huahua Guan, Yongzhu Liang, Xiaodong Fang, Xuanting Jiang, Lijuan Han, Li Huang, Zhimin Yang

**Affiliations:** ^1^State Key Laboratory of Dampness Syndrome of Chinese Medicine, The Second Affiliated Hospital of Guangzhou University of Chinese Medicine, Guangzhou, China; ^2^Guangdong Provincial Hospital of Chinese Medicine, Guangzhou, China; ^3^Zhuhai Branch of Guangdong Provincial Hospital of Chinese Medicine, Zhuhai, China; ^4^Department of Scientific Research, Kangmeihuada GeneTech Co., Ltd., Shenzhen, China

**Keywords:** traditional Chinese medicine constitution, deficient constitution, anemia, gut microbiome, serum metabolomics

## Abstract

**Background:**

Constitution is a valuable part of traditional Chinese medicine theory; it is defined as the internal foundation for the occurrence, development, transformation and outcome of diseases, and has its characteristic gut microbiota. Previous study showed that deficiency constitution was related to lower Hb counts. However, no research has examined how alterations in the gut microbiome induced by deficiency constitution may increase the tendency for anemia.

**Methods:**

We used a multiomics strategy to identify and quantify taxonomies and compounds found under deficient constitution individuals and further explore the possible pathological factors that affect red blood cell indices.

**Results:**

① People with deficient constitution showed lower hemoglobin (Hb), more Firmicutes, less Bacteroidetes, and higher α diversity. ② We identified *Escherichia coli*, *Clostridium bolteae*, *Ruminococcus gnavus*, *Streptococcus parasanguinis* and *Flavonifractor plautii* as potential biomarkers of deficient constitution. ③ *Slackia piriformis*, *Clostridium_sp_L2_50* and *Bacteroides plebeius* were enriched in balanced-constitution individuals, and *Parabacteroides goldsteinii* was the key bacterial marker of balanced constitution. ④ *Flavonifractor plautii* may be a protective factor against the tendency for anemia among deficient individuals. ⑤ *Ruminococcus gnavus* may be the shared microbe base of deficiency constitution-related the tendency for anemia. ⑥ The microorganism abundance of the anaerobic phenotype was lower in deficient constitution group. ⑦ Alterations in the microbiome of deficient-constitution individuals were associated with worse health status and a greater risk of anemia, involving intestinal barrier function, metabolism and immune responses, regulated by short-chain fatty acids and bile acid production.

**Conclusion:**

The composition of the gut microbiome was altered in people with deficient constitution, which may explain their poor health status and tendency toward anemia.

## 1 Introduction

Constitution is an important aspect of traditional Chinese medicine theory. Constitution is defined as the internal foundation for the emergence, development, transformation and outcome of diseases. The discipline of traditional Chinese medicine constitution divides people into nine subgroups: balanced, relatively healthy constitution and eight unbalanced constitutions (qi-deficient, yang-deficient, yin-deficient, phlegm-dampness, dampness-heat, blood stasis, qi-stagnation, and inherited special constitution). People in each unbalanced constitution subgroup are susceptible to certain diseases, a classification that helps us choose specific intervention and disease prevention strategies (1).

According to the traditional Chinese medicine theory, people with a balanced constitution are full of energy, fit, and not predisposed to becoming ill. People in the excessive constitution are likely stucked by some harmful substances (phlegm-dampness, dampness-heat, blood stasis or qi) disturbing physical functions, while people in the deficient constitution are likely lack of some important substances (yang-qi or yin-liquid) maintaining physical functions. A literature review of 1,639 clinical studies showed that none of the research found a relation between the balanced constitution and any disease, while 333 kinds of diseases are closely associated with eight unbalanced constitutions. People in the yang-deficient constitution, yin-deficient constitution and qi-deficient constitution groups, are likely to experience diabetes, stroke and hypertension (especially females) (2). Previous study showed that qi-deficient constitution is accompanied by immune dysfunction leading to inability to resist pathogenic microorganisms (3); yang-deficient constitution exhibit deregulation of various genes involved in energy, lipid, and glucose metabolism (4); yin-deficient constitution related to certain differentially expressed genes associated with the hypothalamus-pituitary–adrenal axis, hypothalamus-pituitary-thyroid axis, cyclic nucleoside system, and immune function (5).

Overall, people in the these three groups all present deficiency statuses of physical and functional, related immunocompromised and hypometabolism (6, 7). One study showed that creatinine (CRE), thyroid-stimulating hormone (TSH), red blood cells (RBCs) and hemoglobin (Hb) levels were lower in people with yang-deficient constitution than in those without (8). Another study of 60 female volunteers aged 35–49, showed that the values of triglyceride (TG), Hb, platelet distribution width (PDW), mean platelet volume (MPV), red cell distribution width standard deviation (RDW-SD) and platelet/larger cell ratio were lower in the yin deficiency group, while the values of white blood cell (WBC) count and RDW coefficient of variation (RDW-CV) were higher in the balanced group (9). Coincidentally, we also found lower Hb counts in individuals with a deficient constitution based on the data from regular physical examinations among hospital staff, and most of these individuals were female.

The World Health Organization defines anemia as a condition in which the number of RBCs or the Hb concentration within them is lower than normal. In adults, anemia is defined as Hb levels of <12.0 g/dL in women and < 13.0 g/dL in men (10). Anemia can be categorized into three types: decreased production, increased destruction and loss of red blood cells through bleeding. All types of anemia result in impaired oxygen delivery to tissues, causing symptoms like weakness and fatigue. The causes of anemia are often multifactorial. Nutritional deficiencies are the most common risk factors for anemia in developing countries. These can result from insufficient dietary intake, increased nutrient losses (e.g., blood loss from parasites, childbirth hemorrhage, or heavy menstrual bleeding), damaged absorption (e.g., lack of intrinsic factor for vitamin B12 absorption, high phytate intake, or *Helicobacter pylori* infection affecting iron absorption), or altered nutrient metabolism (e.g., vitamin A or riboflavin deficiency impaired iron mobilization) (11, 12).

The results of RBC indices are used to diagnose different types of anemia, and each type has a different effect on the size, shape, and/or quality of red blood cells (13). Specifically, mean corpuscular volume (MCV), mean corpuscular hemoglobin (MCH), and mean corpuscular hemoglobin concentration (MCHC) were earliest purposed by Wintrobe in 1929 to define the size and hemoglobin content of red blood cells. If people with larger MCV and lower MCH than normal, it may indicates iron deficiency anemia. If people with smaller MCV and higher MCH than normal, it may indicates vitamin B deficiency related anemia. If people with larger MCV and lower MCHC than normal, it may indicates Thalassemia. If people with higher MCHC than normal, it may indicates hemolytic anemia or hereditary spherocytosis (14). Red cell distribution width (RDW) can be used to quantify the variation in the size of red cells. If people with increased RDW, it may indicates nutritional anemia and sideroblastic anemia. Hematocrit (HCT) is the proportion of blood volume occupied by packed RBC. For many practical approaches, a decrease in HCT is considered equivalent to a decreased Hb concentration (15). These aforementioned values categorized as red blood cell indices are all related to different etiology of anemia, so we assumed people with or without anemia but with one of abnormal red blood cell indices (including Hb, RBC, MCV, MCH, MCHC, RDW and HCT) may have the tendency for anemia in this study.

Over recent years, research on the traditional Chinese medicine and its regulation of intestinal microbiome to improve the symptoms of disease is very extensive (16–19). Different constitution subgroups in Chinese medicine have not only specific clinical characteristics but also characteristic gut microbiota and pathological bases (1, 20–23). A previous report indicated that the α diversity of balanced constitution individuals was considerably higher than that of qi-deficient constitution individuals and the contrast of the β diversity was notably detected between them, having 122 and 4 bacterial taxa that were significantly overactive, respectively. The operational potential of qi-deficient constitution bacterial taxa was decreased in butanoate metabolism and fatty acid metabolism (23). Another randomized clinical trial demonstrated that yang-deficient constitution samples exhibited significantly lower species and showed an increased abundance of Bacteroidetes and Bacteroides, as well as increased levels of gut microbial-derived urinary metabolites, compared to balanced-constitution samples (21).

The intestinal environment is complicated and dynamic, and it significantly influences the host’s constitution. As the lumen of the colon lacks oxygen, anaerobic microbes, such as Bacteroides, Clostridium, and Ruminococcus, are its main occupants, but the numbers of oxygen-tolerant facultative anaerobes and anaerobes increase during dysbiosis (24, 25). Aplastic anemia (26), gestational anemia (27, 28), iron deficiency anemia (29) and cancer-related anemia (30) have been associated with changes in the gut microbiome. On the basis of these conclusions, we hypothesized that changes in the intestinal anaerobic environment may be related to deficient constitution-related increase tendency for anemia.

Therefore, we hypothesized that women with deficient constitution have different microbial compositions and structures from those with a balanced constitution, which predisposes them to specific diseases, such as anemia, associated with intestinal anaerobic environmental disorders.

## 2 Materials and methods

### 2.1 Subjects

The study was conducted at Guangdong Provincial Hospital of Chinese Medicine, Fangcun Branch, Guangzhou, China, among internal clinical staff. This study conformed to the Declaration of Helsinki and the protocol was officially agreed upon the Ethics Committee of the Guangdong Provincial Hospital of Chinese Medicine (Ethical review number: B2017-199-01) and was conducted according to approved guidelines and regulations. All subjects offered verbal informed consent and were able to quit at any time. Data privacy was protected throughout this study.

### 2.2 Design and study population

The hospital staff members underwent a health assessment at the hospital, involving medical history, physical examination, blood hematology, biochemistry analyses and traditional Chinese medicine constitution assessment. The initial details included sex and age. Systolic blood pressure (SBP), diastolic blood pressure (DBP), height, weight, waist circumference and hip circumference were also collected. We used the Constitution in Chinese Medicine Questionnaires (CCMQ) to identify traditional Chinese medicine constitution (31). People with clinical conditions of yang deficiency, yin deficiency and qi deficiency according to the CCMQ responses were categorized as the deficient constitution group (DCG), while people without any clinical conditions of basis constitutions were cataloged as the balanced constitution group (BCG). Fifty-two clinical features were assessed in this study. According to the results Hb, RBC, HCT, MCV, MCH, MCHC and RDW, people were also divided into a normal group (NG) and an abnormal group (AG) if one of the indices was out of the normal range. We assumed people with one of abnormal red blood cell indices (including Hb, RBC, MCV, MCH, MCHC, RDW and HCT) may have the tendency for anemia in this study. As for sample size, a convenience sample was chosen because it was beyond the scope of the study to include a statistically powered sample size due to the limitation of resources.

#### 2.2.1 Inclusion criteria of participants

① Age ≥ 18 years and ≤ 60 years; ② Female; ③ Underwent a health assessment in the hospital, including medical history, physical examination, blood hematology, biochemistry analyses and traditional Chinese medicine constitution assessment; ④ Categorized as the deficient constitution (including yang deficiency, yin deficiency and qi deficiency) or balanced constitution according to the CCMQ; ⑤ The informed consent shall be agreed by the participants.

#### 2.2.2 Exclusion criteria of participants

① Age < 18 years or > 60 years; ② Male; ③ During menstruation, pregnancy, child birth and/or baby nursing period; ④ Accompanied by hematological malignancies or other diseases that could significantly affect survival; ⑤ Categorized as the excesssive constitution (including phlegm-dampness, dampness-heat, blood-stasis, and qi-depression) or special-diathesis constitution according to the CCMQ; ⑥ Used antibiotics in last 6 months.

Finally, 129 participants, including 23 individuals in the deficient constitution associated with the DCAG, 56 individuals in the DCNG, 15 individuals in the BCAG and 35 individuals in the BCNG, were selected. A total of 127 fecal and serum samples were collected.

### 2.3 Traditional Chinese medicine constitution assessment

Considering its validity and reliability, the CCMQ was used to assess traditional Chinese medicine constitution, which comprises 9 subscales with a total of 60 questions, of each scored from 1 (none) to 5 (always), evaluating the constitution type by adding the item scores based on Wang Qi’s nine-point method of constitution classification. This method divides human constitution into nine types, among which balanced constitution is the healthiest type, while the other eight types are pathological types hinting at unsatisfactory status (31). People with qi-deficient, yang-deficient, and yin-deficient constitutions are deficient, while those with phlegm-dampness, dampness-heat, blood-stasis, and qi-depression constitutions are excesssive.

### 2.4 Blood biochemical analysis

This study assessed 52 biomarkers as the clinical features: albumin (ALB), globulin (GLB), albumin/globulin (ALB/GLB), alanine transaminase (ALT), aspartate transaminase (AST), aspartate transaminase/alanine transaminase (AST/ALT), serum gamma-glutamyl transferase (GGT), alpha fetoprotein (AFP), carcinoembryonic antigen (CEA), creatinine (Cre), urine acid (UA), fasting blood glucose (Glu), serum total cholesterol (TC), TG, high-density lipoprotein cholesterol (HDLC), nonhigh-density lipoprotein cholesterol (non HDLC), low-density lipoprotein cholesterol (LDLC), serum total bile acid (TBA), TSH, C-reactive protein (CRP), anti-cyclic peptide containing citrulline (anti-CCP), Epstein–Barr virus nuclear antigen immunoglobulin A (NA IgA), Epstein–Barr virus viral capsid antigen immunoglobulin A (VCA IgA), procalcitonin (PCT), functional thyroid 3 (FT3), free triiodothyronine (FT3), free thyroxine (FT4), total protein (TP), urea, estimated glomerular filtration rate (eGFR), interleukin-27 (IL27), RBC, WBC count, Hb, HCT, MCV, MCH, MCHC, platelet (PLT), neutrophil (NEUT), percentage of neutrophils (NEUT%), lymphocyte (LYM), percentage of lymphocyte (LYM%), monocyte (MONO), percentage of monocyte (MONO%), eosinophil (EOSIN), percentage of eosinophil (EOSIN%), basophil (BASO), percentage of basophil (BASO%), Meam platelet volume (MPV), RDW and platelet distribution width (PDW).

### 2.5 Fecal sample collection, sequencing and microbial data analysis

Stool samples of at least 2 g were saved, placed in arid tubs and stored at −80°C before gDNA extraction. After isolation, the DNA was sonicated for fragmentation, resulting a size of 350 bp. The library was prepared by using the TruSeq DNA HT Sample Prep Kit. Suitable libraries were screened and sequenced by a paired-end strategy with the Illumina HiSeq 2000 platform. After filtering out low-quality and host derived sequences, metagenomics bioinformatics pipeline was performed according to the bioBakery3 (32). After preprocessing, there were around 6 G of clean data for each sample. We used MetaPhlan2 to obtain the taxonomy of the metagenomes and applied HUMAnN2 to characterize the microbial gene and biochemical pathways (33, 34). The bacterial α diversity was evaluated by the Shannon index, observed species, and simpson index. Differences in microbial characteristics were examined by the Wilcoxon rank-sum test and linear discriminant analysis effect size (LEfSe) (35).

### 2.6 Serum sample collection, sequencing and metabolic data analysis

Blood and stool samples were taken from each volunteer. The 100 μL serum of every sample was positioned in a 96-well plate and mixed by 300 μL of precooled extract solution (methanol: acetonitrile, 2:1, v/v). After brief vortex, the samples were incubated for 2 h at −20°C, centrifuged at 4000 rpm for 20 min, freeze-dried, redissolved in 150 μL of 50% methanol, and then centrifuged at 4000 rpm for 30 min. One hundred microliters of sample was drawn out aim for metabolite extraction. A quality control sample was made by combining equal amounts of supernatants from all samples. LC–MS/MS analysis was conducted using the UHPLC system (Vanquish, Thermo Fisher Scientific) coupled to a Q Exactive HFX mass spectrometer (Orbitrap MS, Thermo). After converting the raw data to mzXML format, peak detection, extraction, alignment and integration were performed sequentially using ProteoWizard. PCA and OPLS-DA models were used to discriminate the classifications for different groups in score plots. Then, based on OPL-DA loading plots, we selected ions with VIP > 1.5 to identify significant metabolites in each group. Next, univariate analysis of t test and fold-change were used for those notable distinctive ions with VIP > 1.5 and fold-changes > 1.5 (or < 0.67). They were recognized and interpreted according to studies of their exact masses in metabolomic-associated databases: METLIN,^1^ HMDB^2^ and KEGG.^3^ Metabolomics bioinformation analysis was performed by MetaboAnalyst software.^4^

### 2.7 Statistical analysis

All data were double recorded to guarantee correct entry. The fundamental characteristics of the objects were reported by counts and abundances. Statistical analysis was performed using the R statistical package (v.4.0.4). The vegan package was used for microbial community ecology analysis, and the distribution-free test, Kruskal–Wallis test, Wilcoxon test, and Spearman correlation test were uesd for relative abundance analysis. Factor analysis and one-way analysis of variance were used for normally distributed data. MaAsLin 2(Microbiome Multivariable Association with Linear Models) was conducted to explore the association between metobolites as an independent variable and the count of clinical biomarkers (including deficiency constitution group, age, BMI, MCHC, MCH, MCV, RDW, HCT, RBC, Hb counts) as the dependent variable or covariates. *P* < 0.05 was defined as statistically significant.

### 2.8 Integrative multi-omic analysis

Procrustes analysis was performed to examine the congruence of the two-dimensional shapes generated by PCAs between metabolomics and microbiome by R vegan package.

Two-way Orthogonal Partial Least Squares analysis (O2PLS) performed by R OmicsPLS package was used to reveal the relationship between microbiota at species level and metabolites, in which, microbiota data were mapped to metabolites on low dimensional hyper planes. Microbiota with larger VIP value (>1), are the most related to explain metabolites. The correlation matrix display the pair-wise correlation between all variables.

MetOrigin was uesd to trace the origins of bacteria and identify their certain metabolic reactions involved for understanding the complicated interaction between microbiome and metabolites. It was performed on its authorized website.^5^

Stepwise mediation analysis performed by R mediate package was conducted for multi-omic modules along the microbiome-metabolite-host axis to assess the relationship of the constitution related-microbiome with the tendency for anemia, with Hb, RBC, HCT, MCV, MCH, MCHC and RDW being a continuous dependent variable, and the tendency for anemia being a discrete dependent variable.

## 3 Results

### 3.1 Summary of clinical characteristics

A total of 129 female hospital staff members were included in our study, including 35 people in the balanced constitution mormal group (BCNG), 15 people in the balanced constitution abnormal group (BCAG), 56 people in the deficient constitution normal group (DCNG) and 23 people in the deficient constitution abnormal group (DCAG). Compared to the broad balanced constitution group (BCG), the broad deficient constitution group (DCG) had lower Hb, mean corpuscular hemoglobin concentration (MCHC), systolic blood pressure (SBP), and total bile acids (TBA) values, tending toward anemia characteristics. There was no significant difference in other indicators between the two groups including age (Table 1).

**Table 1:** Clinical Characteristics of the DCG and BCG.

Characteristics			
Characteristic	Balance, *N* = 50^a^	Deficiency, *N* = 79^a^	*p*-value^**b**^
Red blood cell indices (counts)			>0.99
Abnormal	15 (30%)	23 (29%)	
Normal	35 (70%)	56 (71%)	
Groups (counts)			<0.001
BalanceAbnormal	15 (30%)	0 (0%)	
BalanceNormal	35 (70%)	0 (0%)	
DeficiencyAbnormal	0 (0%)	23 (29%)	
DeficiencyNormal	0 (0%)	56 (71%)	
Age (yrs)	34 (6)	35 (6)	0.32
BMI (Body mass index, kg/m^2^)	21.62 (3.34)	21.01 (2.57)	0.27
SBP (Systolic blood pressure, mmHg)	113 (8)	109 (12)	0.027
DBP (Diastolic blood pressure, mmHg)	69 (6)	68 (8)	0.43
GLU (Blood glucose, mmol/L)	5.20 (0.76)	5.15 (0.55)	0.68
LDLC (Low density lipoprotein, mmol/L)	2.85 (0.68)	2.77 (0.62)	0.46
TC (Total cholesterol, mmol/L)	4.75 (0.77)	4.65 (0.71)	0.46
TG (Triglyceride, mmol/L)	0.91 (0.37)	0.88 (0.37)	0.67
HDLC (High density lipoprotein cholesterol, mmol/L)	1.53 (0.25)	1.50 (0.32)	0.5
CRP (C-reactive protein, mg/L)	1.08 (2.00)	1.03 (2.35)	0.88
NEUT (Neutrophil, 10^9^/L)	3.50 (1.37)	3.32 (1.23)	0.46
MONO (Monocyte, 10^9^/L)	0.38 (0.11)	0.37 (0.11)	0.92
EOSIN (Eosinophil. 10^9^/L)	0.12 (0.10)	0.11 (0.08)	0.47
MCHC (Mean corpuscular hemoglobin concentration, g/L)	330 (9)	326 (12)	0.02
MCH (Mean corpuscular hemoglobin, pg)	29.1 (2.4)	28.9 (3.2)	0.67
MCV (Mean corpuscular volume, fL)	88 (6)	89 (8)	0.75
MPV (Meam platelet volume, fL)	10.03 (0.95)	10.32 (0.87)	0.091
LYM (Iymphocyte, 10^9^/L)	1.98 (0.52)	1.82 (0.48)	0.086
WBC (White blood cell, 10^9^/L)	6.01 (1.62)	5.66 (1.53)	0.22
RDW (Red blood cell distribution width, %)	12.47 (1.04)	12.63 (1.38)	0.45
HCT (Hematocrit, %)	40.11 (2.64)	39.25 (2.36)	0.065
RBC (Red blood cell, 10^12^/L)	4.57 (0.47)	4.47 (0.49)	0.23
PDW (Platelet distribution width, fL)	11.44 (2.08)	11.78 (2.34)	0.39
PCT (Procalcitonin, ng/mL)	0.27 (0.04)	0.26 (0.05)	0.51
PLT (Platelet, 10^9^/L)	269 (50)	254 (57)	0.12
Hb (Hemoglobin, g/L)	132 (10)	128 (9)	0.01
TBA (μmol/L)	6.20 (7.15)	3.65 (2.27)	0.018
TP (Total protein, g/L)	74.8 (4.0)	74.7 (3.6)	0.82
IL27 (Interleukin-27, pg./mL)	697 (1,281)	566 (1,276)	0.57

^a^*n* (%); Mean (SD). ^b^Fisher’s exact test; Welch Two Sample *t*-test.

### 3.2 Differences in community composition of the gut microbiota

**Figure 1:**
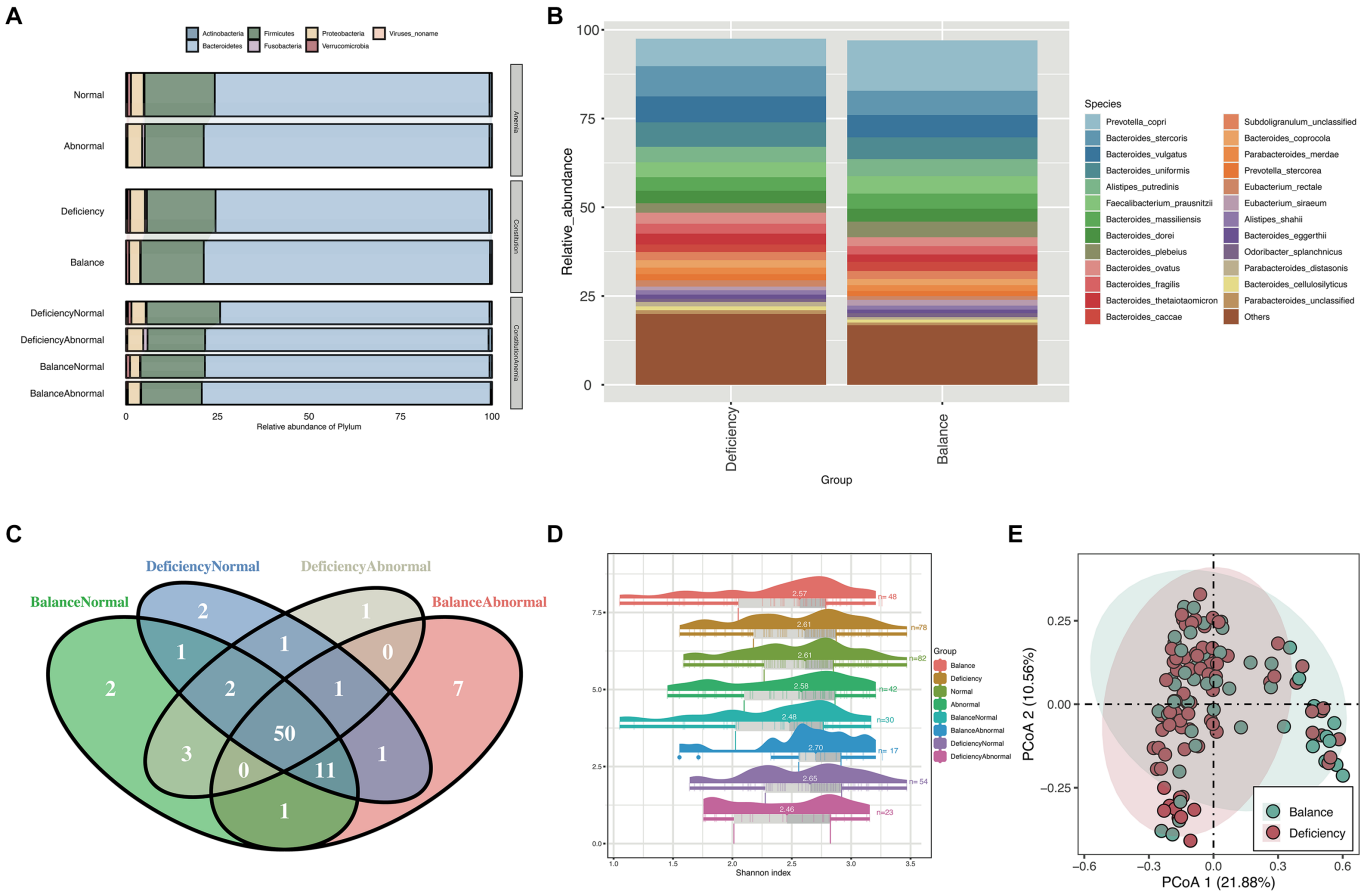
Taxonomy composition and diversity analysis. **(A)** Stacked bar chart for the relative abundance of phylum between different groups and **(B)** stacked bar chart for the relative abundance of species between the BCG and the DCG. Different colors represent different bacteria. **(C)** Venn diagram of species counts (median value) in the DCNG, DCAG, BCNG and BCAG. **(D)** Raincloud plots of Shannon indices for the α-diversity analysis in the BCG, DCG, normal blood indices group (NG), abnormal blood indices group (AG), DCNG, DCAG, BCNG and BCAG. **(E)** PCoA analysis for β diversity analysis in DCG and BCG based on Bray–Curtis distance.

To distinguish the relative abundances of gut microbiota between groups, we examined the taxonomic composition in gut microbiota of different group samples at the phylum level (Figure 1A), and further explored the differential taxonomic composition of the DCG and BCG at the species level (Figure 1B). Bacteroides and Firmicutes were the two dominant categories, but there was more Firmicutes and fewer Bacteroidetes in the DCG than in the BCG.

The Venn diagram showed that 50 species were shared between the DCNG, DCAG, BCNG and BCAG, 2 species were unique to the DCNG, 1 species was unique to the DCAG, 2 species were unique to the BCNG, and 7 species were unique to the BCAG (Figure 1C).

Then, we analyzed the α diversity of all groups based upon pairwise comparisons by the Wilcoxon rank-sum test (Figure 1D). Generally, no significant differences were found in the α diversity based on the Shannon index of the microbiota (*p* > 0.05). We also analyzed their β diversity. Based on the PCoA results, a contrast in the gut microbiota was detected between the DCG and BCG samples (Figure 1E).

### 3.3 Differences in biological markers of the gut microbiota

**Figure 2:**
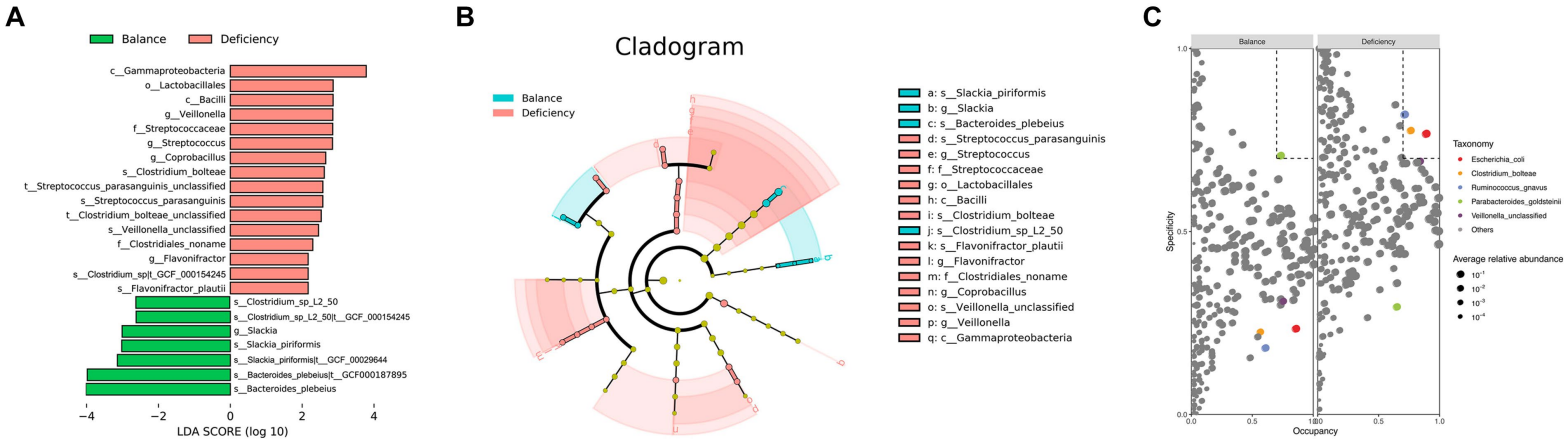
Taxonomy differences and species dominance analysis. **(A)** Bar chart of LDA logarithmic scores of biological markers spotted by LEfSe (*p* < 0.05 and LDA cutoff >2.0) for all levels of features differentially abundant between DGC and BCG. **(B)** Cladogram representing the differences by LEfSe between the DCG and the BCG. Colors distinguish between BCG (blue) and DCG (red), and the size of the dot relates to its relative and logarithmically scaled abundances. **(C)** SPEC-OCCU analysis plot to identify the specialist species between the DCG and the BCG (occupancy>70%, specificity>70%).

To identify characteristic gut microbiota in the DCG and BCG, we analyzed bacterial markers by LEfSe analysis to distinguish people between them on the grounds of the relative abundance of each taxon (Figure 2A). The cladogram displays the phylogenetic distribution between the DCG and the BCG from the phylum to the genus-level characteristic organisms. It highlights microbial subtrees that were differentially abundant by LEfSe, and their effect sizes as assessed by linear discriminant analysis (Figure 2B). As shown in the figure, there was a greater abundance of the *Slackia piriformis, Clostridium_*sp_L2_50, and *Bacteroides plebeius* species in the BCG. There was a greater abundance of *Flavonifractor plautii* (family Clotridiaceae), *Streptococcus parasanguinis* (family Streptococcaceae), ordor Lactobacillales, class Bacilli, as well as Gammaproteobacteria, Veillonella and Coprobacillus, and *Clostridium bolteae*, in the DCG. Overall, significant differences were found in both species abundance and composition between the two groups of samples at the class, order, family, genus, species and strain levels.

Based on specificity occupancy (SPEC-OCCU) analysis, *Escherichia coli*, *Clostridium bolteae* and *Ruminococcus gnavus* were distinguished as dominant bacterial markers of the DCG, while *Parabacteroides goldsteinii* was considered the dominant bacterial marker in the BCG (Figure 2C).

The same method was used for LEfSe analysis to distinguish between people with tendency for anemia and relatively healthy people. *Sutterella wadsworthensis* had a notably higher abundance in the AG, while *Megasphaera unclassified* and *Clostridium asparagiforme* were higher in the NG (Supplementary Figure S1A). Taken together, the data showed that significant differences were present in both species abundance and composition between the AG and the NG at the species level, but they were incompatible with the characteristics of gut microbiota in the DCG and the BCG. This suggests that differential gut microbiota in traditional Chinese medicine constitution may not be directly associated with the risk of anemia, and not all the people with deficient constitution progress to anemia.

To reveal the role of the microbiota in the pathological process of anemia, we measured bacterial markers in the four groups (DCNG vs. DCAG vs. BCNG vs. BCAG). At the species level, the abundances of *Streptococcus australis* and *Flavonifractor plautii* were markedly higher in the BCAG, while the abundance of *Clostridium leptum* was notably higher in the DCNG than in the other groups (Supplementary Figure S1B). To explore how constitution factors influence people who are prone to anemia, we quantified the differentially expressed microbiota between DCs and BCs in normal or abnormal situations. For comparisons between the DCNG and the BCNG, the differential analysis at the species level displayed markedly higher abundance of *Bacteroides thetaiotaomicron*, *Streptococcus parasanguinis*, *Clostridium bolteae*, *Streptococcus australis*, *Flavonifractor plautii*, *Streptococcus salivarius* and *Lachnospiraceae* bacterium_7_1_58FFA in the DCNG, while there was a higher abundance of *Peptostreptococcaceae* noname unclassified and *Anaerotruncus* unclassified in the BCNG (Supplementary Figure S1C). Notably, *Flavonifractor plautii, Streptococcus parasanguinis* and *Clostridium bolteae* matched the results of differences in gut microbiota between the DCG and the BCG. To compare the results between the DCAG and BCAG, the differential analysis at the species level displayed markedly higher abundances of *Bacteroides vulgatus*, *Ruminococcus gnavus*, *Parabacteroides* unclassified and *Coprobacillus* unclassified in the DCAG, while the abundances of *Akkermansia muciniphila*, *Eubacterium eligers*, *Alistipes finegens*, *Bacteroides nordii*, *Parabacteroides goldsteinii*, *Clostridium leptum*, *Subdobigranulum* unclassified and *Bacteroides plebeius* were notably higher in the BCAG (Supplementary Figure S1D). Notably, *Bacteroides plebeius* and *Parabacteroides goldsteinii* were characteristic gut species distinguishing the DCG from the BCG.

**Figure 3:**
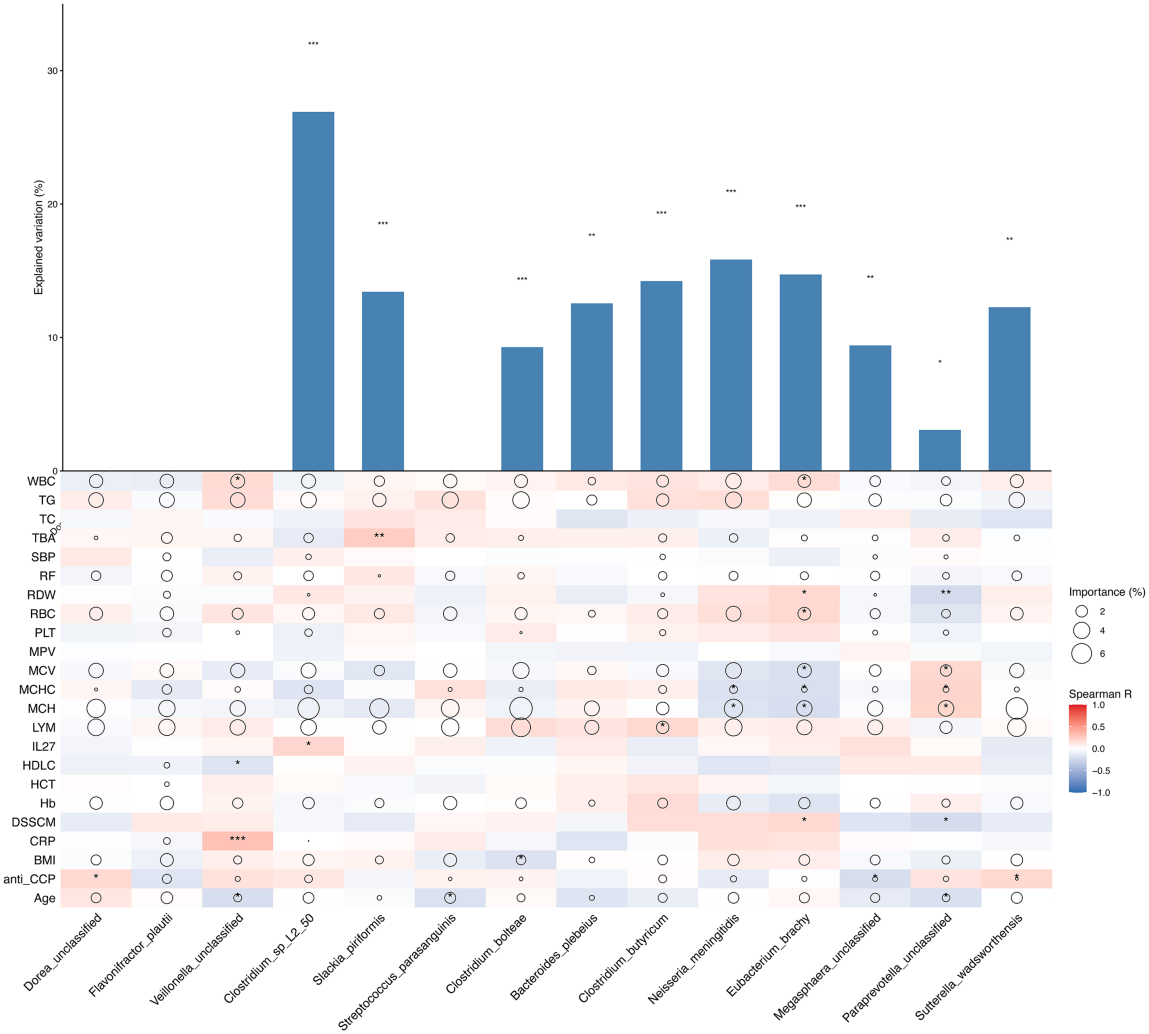
Contribution of phenotype to species relative abundance differences on the basis of correlation analysis and a random forest model between different groups (BCG vs. DCG; AG vs. NG; DCNG vs. DCAG vs. BCNG vs. BCAG). The size of each circle denotes the variable importance [calculated by means of multiple regression modeling and variance decomposition analysis (Maaslin2)]. Colors denote the strength of the Spearman correlation coefficients.

To distinguish the association between clinical characteristics and the microbial society structure, we inspected the contributions of clinical characteristics to the variety of microbial societies and differences in the relevant abundances of microbial species on the basis of the correlation coefficient and multiple regression model (Figure 3), and the major predictors were identified. Statistical significance was found for microbial species of *Paraprevotella* unclassified (*p <* 0.05), and stronger statistical significance was found for *Sutterella wadsworthensis*, *Megasphaera* unclassified and *Bacteriudes plebeius* (*p <* 0.01), while extreme statistical significance was found for *Clostridium*_sp_L2_50, *Slackia piriformis*, *Clostridium butyricum*, *Neisseria meningitidis* and *Eubacterium brachy* (*p <* 0.001). Anemia-related indices, including MCV, MCH, Hb, and RBC, represented the most variable importance to the bacterial species mentioned above. Immunity indicators, including WBC, LYM, and TG, also contributed to the explanatory degree of the variance change in gut microbiota. The heatmap reflects the correlation between primary clinical characteristics and different characteristic species in the comparison of different groups (BCG vs. DCG; AG vs. NG; DCNG vs. DCAG vs. BCNG vs. BCAG).

**Figure 4:**
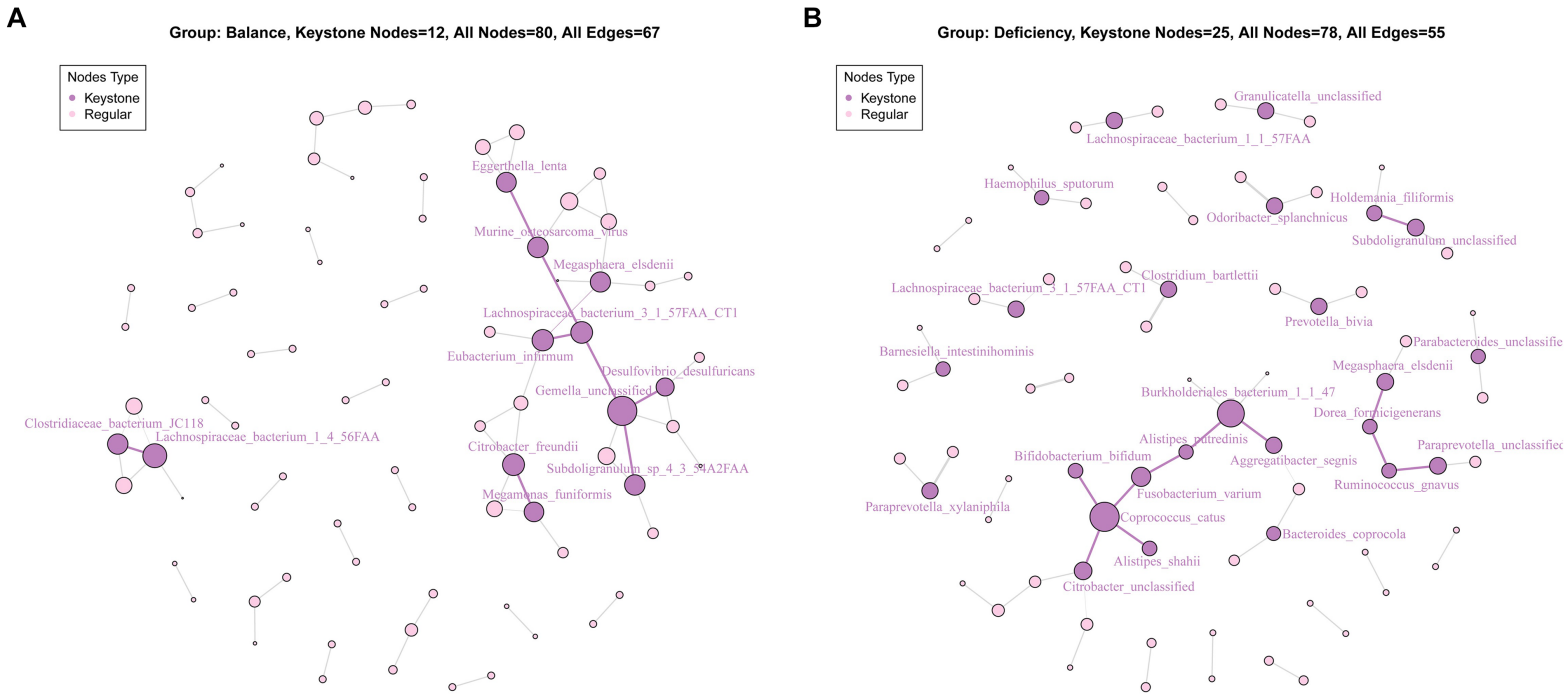
Key species analysis based on community interaction networks in the BCG **(A)** and DCG **(B)**. The lines between species indicate correlations: red bold edge means negative correlation; black weak edge means positive correlation. The size of a node means the degree of importance in the network; the dark purple nodes show the keystone species.

For the communication of bacteria, we performed network analysis of the bacteria of the DCG and the BCG. Although the numbers of the keystone species in the DCG was more than that in the BCG, there were stronger networks among characteristic bacterial communities in the BCG (Figure 4A), and more independent communities in the DCG (Figure 4B).

### 3.4 Differences in metagenomic functional pathways

**Figure 5:**
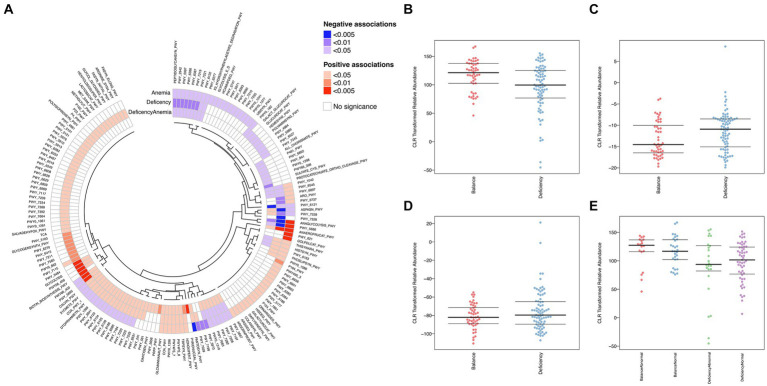
Differentially functional microbiota in different groups of samples. **(A)** Heatmap and circular dendrogram of the microbial, describing the notable enrichment (red) or notable underrepresentation (blue) in each comparison (deficiency: DCG vs. BCG; anemia: NG vs. AG; deficiency anemia: BCNG vs. DCAG). **(B–E)** BugBase analysis for organism-level microbiome phenotypes, including anaerobic **(B)**, aerobic **(C)**, and facultative-anaerobic **(D)**, between the DCG and the BCG samples and **(E)** anaerobic features among the DCNG, DCAG, BCNG and BCAG.

To examine the possible operation of the intestinal microbial society and their relationship in deficient constitution-related the tendency for anemia, we analyzed the relevant abundance of 339 metabolic pathways based on the UniRef90 database (Figure 5A). Of note, 252 (74%) of the pathways were related to at least one comparison in groups.

Compared to the BCG, seven pathways were extremely significantly upregulated in the DCG (*p* < 0.005). They were PWY_5484 (glycolysis II, from fructose 6-phosphate), GLYCOLOLYSIS, PWY66_400 (glycolysis VI, metazoan), PWY_409 (superpathway of purine nucleotide salvage), ANAEROFRUCAT_PWY (homolactic fermentation), BIOTIN_BIOSYNTHESIS_PWY, and PWY_5695 (urate biosynthesis/inosine 5′-phosphate degradation). Most of these pathways were associated with energy metabolism, especially the glycolytic cycle. Comparisons of DCG vs. BCG and AG vs. NG, also showed a difference in ANAEROFRUCAT_PWY (homolactic fermentation), a process of transformation from sugars to cellular energy and the metabolic byproduct lactate. Based on the threshold of *p* < 0.05, more shared pathways were observed in the comparisons of DCG vs. BCG, AG vs. NG and BCAG vs. BCNG vs. DCNG vs. DCAG. In each pair, the activity of 10 pathways was downregulated in the former group (including PEPTIDOGLYCANSYN-PWY, PWY-2942, PWY-5097, PWY-6386, PWY-6387, PWY-7219, PWY-7221, PWY-6122, PWY-6277 and PWY-6121, which are mainly related to nucleotide biosynthesis and peptidoglycan biosynthesis), while 8 were upregulated (including NAEROFRUCAT-PWY, NONOXIPENT-PWY, TRPSYN-PWY, PWY4FS-8, PWY4FS-7, PWY0-1296, COA-PWY and GLCMANNANAUT-PWY, which are mainly related to survival of *Escherichia coli* and multiroute energy metabolism). These results indicated the pathological basis of deficient constitution-related increases the tendency for anemia and emphasized *Escherichia coli* as the dominant bacterial marker in the DCG.

We then applied BugBase to predict organism-level microbiome phenotypes between the DCG and the BCG. The results showed that the abundance of microorganism with an anaerobic phenotype was higher in the BCG than the DCG, while the abundance of microorganism with aerobic and facultative-anaerobic phenotypes was lower in the BCG (Figures 5B–D). More specifically, the anaerobic phenotype was most abundant in the BCAG, followed by BCNG, DCNG and DCAG in descending ordor (Figure 5E).

### 3.5 Identification of differentially abundant metabolites

**Figure 6:**
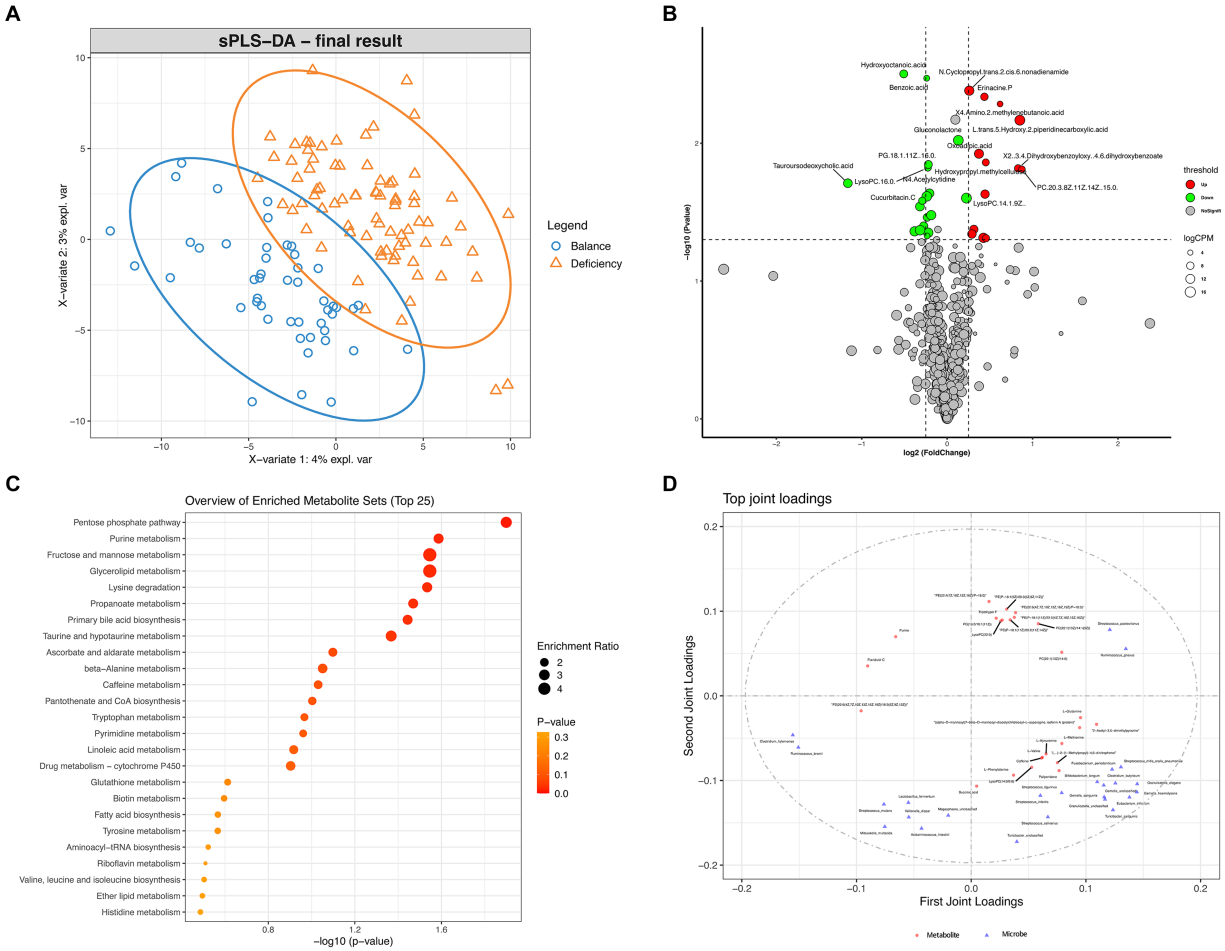
Differential analysis of metabolome features. **(A)** PLS-DA analysis of metabolites between DCGs and BCGs. **(B)** Volcano plot of differential metabolites between the DCG and the BCG. **(C)** Scatter plot for functional enrichment analysis of differential metabolites between the DCG and the BCG. **(D)** O2PLS analysis of microbiota metabolite relationships.

Differentially aboundant metabolites in the serum of DCG and BCG individuals were recognized by metabolomic profiling. Score plots of the PLS-DA models showed discrimination between them in the serous metabolic profile (Figure 6A). Differential metabolites are displayed in the volcano plot (Figure 6B), and we noticed 13 metabolites that were significantly upregulated (including oxoadipic acid, etc.) and 9 metabolites that were significantly downregulated (including cucurbitacin C, decanoylcarnitine, tauroursodeoxycholic acid, glycerol triundecanoate, etc.) in the serum of DCG individuals (*p* < 0.05). The same method was applied to discover differential metabolites in the serum of AG individuals, and 14 metabolites were upregulated (inclusive of 2-hydroxybutyric acid, L-methionine, etc.), while 33 metabolites were notably downregulated (including cucurbitacin C and cholic acid, etc.; *p* < 0.05; Supplementary Figure S2).

To explore the potential correlations between the changes in clinical index and metabolomic features, we used spearman’s correlation coefficients to measure the linear relationship between clinical index and metabolomic features. There are some significant correlation was displayed between them (Supplementary Figure S3; *p* < 0.05). Considering that these metabolites may be related to confounding factors of aforementioned clinical features, we made linear models with covariate adjustment analysis of bacteria in the DCG (Supplementary Figure S4). The resulting plot compares the *p* values for each metabolite both before (x-axis) and after (y-axis) covariate adjustment. It shows that L-gluconolactone and pentaneitrile were significant only after adjusting for significantly differential clinical features, including age, BMI, MCHC, MCH, MCV, RDW, HCT, RBC and Hb, while gluconolactone and hydroxyoctanoic acid were significant before and after adjustment.

We further used Maaslin2 to find multivariate associations between clinical data and metabolomic features of the bacterium in the DCG, taking deficiency constitution, age, BMI, MCHC, MCH, MCV, RDW, HCT, RBC and Hb into account. Galacturonic acid (Supplementary Figure S5A; FDR < 0.001) and ajocysteine (Supplementary Figure S5B; FDR < 0.001) had a negative linear correlation with Hb, while linoelaidic acid had a positive linear correlation with RBCs (Supplementary Figure S5C; FDR < 0.001).

Then, a scatter plot for functional enrichment analysis of differential metabolites between the DCG and the BCG was used to explore the biological pathways of the DCG (Figure 6C; *p* < 0.05). A total of 65 pathways were significantly enriched, 25 of which were enriched in protein metabolism (including “Lysine degradation,” “beta-Alanine metabolism,” “Tryptophan metabolism,” “Biotin metabolism,” “Tyrosine metabolism,” “Aminoacyl-tRNA biosynthesis,” “Valine, leucine and isoleucine biosynthesis,” and “Histidine metabolism”) and glycolipid metabolism (including “Fructose and mannose metabolism,” “Glycerolipid metabolism,” “Fatty acid biosynthesis,” “Linoleic Acid,” “Biotin metabolism,” “Ether lipid metabolism,” “Primary bile acid biosynthesis,” and “Taurine and hypotaurine metabolism”). The subsequent reaction converts the products to CoA and can be shunted to the TCA cycle (including “pantothenate and CoA biosynthesis” and “pyrimidine metabolism”). Notably, taking the enrichment ratio and *p* value into consideration, the most significant biological pathway was related to the “pentose phosphate pathway,” as the first-line defense against oxidative stress and is critical to cell proliferation, survival, and senescence. “glutathione metabolism” and “ascorbate and aldarate metabolism” were also connected with oxidant stress. The results indicated that complex energy metabolism and oxidative stress alterations are involved in deficient constitution.

To recognize the associations between different members of the intestinal microbiome and metabolites, we assembled a network of cooccurring bacteria and metabolites and interrogated it for modules via Procrustes analysis and O2PLS analysis. Procrustes analysis showed a significant correlation between the microbial community and metabolites in different samples (Supplementary Figure S6; M2 = 0.217, *p* < 0.001). O2PLS analysis revealed the significant and biologically relevant coordination between bateria and metabolites (Figure 6D). Several kinds of phosphatidylcholine (PC, the basic components of the lipid bilayer of cells involved in metabolism and signaling) were significantly correlated with *Ruminococcus gnavus* (the dominant bacterial marker of DCG).

To characterize this intricate interaction between the microbiome and metabolites, we used MetOrigin to distinguish which bacteria contribute and how to specific metabolic reactions by determining where metabolites come from. Taking characteristic metabolites of deficiency constitution and those correlated with Hb and RBC into account, 3 metabolites were from microbiota, 7 metabolites were from both host and bacteria (cometabolism), and others were related to drugs, food or something else (Supplementary Figure S7A). Based on the metabolites involved, 13 pathways were enriched, among which “C5-branched dibasic acid metabolism” (derived solely from microbiota) was most significant and was found to be distributed in the class Gammaproteobacteria (the greatest abundance of differential microbiota in DCG) (Supplementary Figures S7B,C). *Escherichia coli*, as the dominant bacterial marker of DCG based on SPEC-OCCU analysis, was from the class Gammaproteobacteria. Moreover, the class Gammaproteobacteria and class Bacilli (the second most abundant of differential microbe in the DCG) also contributed to the pathways “pentose and glucuronate interconversions” and “ascorbate and aldarate metabolism” (Supplementary Figures S7D,E), while the order Lactobacillales (the third most abundant differential microbe in the DCG) contributed to the pathways “Amino sugar and nucleotide sugar metabolism” (Supplementary Figure S7F). These pathways were all related to galacturonic acid, which had a negative linear correlation with Hb.

To explore the relationship among metabolites, microbial community and tendency for anemia, we used mediation analysis to investigate metabolites as the mediator of the relation between microbial community and tendency for anemia based on their abundances and counts. We discovered L-Glutamine and D-Glutamine mediated the *Bacteroides plebeius*-tendency for anemia association, while alpha−Santalyl phenylacetate mediated the *Flavonifractor plautii*-tendency for anemia association, both in a negative way (Supplementary Figure S8A; *p* < 0.05). As for the tendency for anemia, we also found alpha−Santalyl phenylacetate mediated the *Bacteroides plebeius*-MCHC association in a negative way (Supplementary Figure S8B; *p* < 0.05).

## 4 Discussion

In this study, we described the clinical characteristics of individuals with deficient constitution, and our microbiome study identified the distinctive compositional and functional patterns of their intestinal microenvironments. Based on the negative correlation between deficient constitution and Hb concentration, we suggest that people with deficient constitution are at risk of anemia. Therefore, we divided people into four groups according to traditional Chinese medicine constitution (deficient constitution versus balanced constitution) and the tendency for anemia (based on laboratory-measured Hb counts, RBC, HCT, MCV, MCH, MCHC and RDW), including the BCNG, BCAG, DCNG and DCAG, and we explored conceivable pathological processes of deficient constitution-related increases the tendency for anemia.

Previous research on the gut microbiota of the qi-deficient constitution, yang-deficient constitution and yin-deficient constitution showed a lower Shannon index of α diversity and differential microbiota profiles compared to the balanced constitution. These studies were mainly carried out in the northern part of China, such as Shandong Province and Beijing (3, 36). There were two studies in the southern part of China: one study screened 461 healthy balanced-constitution individuals and 166 qi-deficient individuals from 5,987 college students (23), and another enrolled 461 balanced-constitution and 76 yang-deficient constitution undergraduates aged 18 to 22 (21). In contrast with previous findings, the Shannon index of α diversity of the microbiota from the DCG was higher than that of the microbiota from the BCG at all levels in this study. Humans are omnivorous primates, and their gut microbiota varies widely across geography (37, 38) and age (39, 40). Our study enrolled female hospital staff members (age from 24 to 54 years old, mean = 34.40) in Guangzhou, southern China. We assumed that differences in age, geographical location, lifestyle and long-term diet may have contributed to this unexpected result. The Shannon index reflects the richness and evenness of species, so we concluded that there were more heterogeneous biomes in the DCG than in the BCG.

The species had roughly the same counts between the DCG and the BCG, but there were increased Firmicutes and decreased Bacteroidetes in DCG. In latest reports, a higher Firmicutes-to-Bacteroidetes ratio was spotted in both overweight rats and humans, involving energy metabolism dysfunction (41–43). The bacterial taxon information of participants was identical to that of healthy Asian populations published in earlier studies (44), but the microbiota were unalike between DCG and BCG.

Through LEfSe analysis and SPEC-OCCU analysis, we focused on *Flavonifractor plautii*, *Streptococcus parasanguinis*, *Clostridium bolteae*, *Escherichia coli*, and *Ruminococcus gnavus* as markers specifically enriched in the DCG at the species level. In line with our findings, as a key bacterial marker of DCG, *Escherichia coli*, with higher occupancy and specificity, is a most facultatively anaerobic and opportunistic pathogen. For *Clostridium bolteae,* its abundance was mostly attributed to MCH, LYM, MCV, TG, WBC counts and RBC counts in this study, indicating that *Clostridium bolteae* might be connected with anemia, immunity and lipid metabolism. It is usually considered an opportunistic pathogen (45) that is associated with some kinds of rare autoimmune diseases, such as immunoglobulin G4-related disease, systemic sclerosis and neuromyelitis optica spectrum disorders (46, 47). A previous study demonstrated that *Flavonifractor plautii* treatment calmed antigen-stimulated Th2 immune responses, consequently restraining interleukin (IL)-4 and ovalbumin-specific IgE production in ovalbumin-sensitized mice (48). Another study showed that Gd-IgA1-associated α-galactosidase and α-N-acetylgalactosaminidase from *Flavonifractor plautii* were conspicuous in IgA nephropathy patients. Thus, *Flavonifractor plautii* has an impact on the host immune system. On the other hand, *Flavonifractor plautii*, found to be depleted in the microbiota of obese subject, has been investigated as a putative biomarker of healthy status, related to butyrate and propionate (48–50). Comparison of the four groups showed that *Flavonifractor plautii* was not only the characteristic bacteria enriched in the DCG but also a significant species in the DCNG, and was negative associated with the tendency for anemia mediated by alpha−Santalyl phenylacetate, which indicates that it may be a beneficial factor that helps some deficient-constitution individuals avoid anemia. People with a deficient constitution are characterized by hypoimmunity and low energy (6). These individuals also had increased activity in pathways associated with energy metabolism, especially the glycolytic cycle, based on the UniRef90 database of microbiota in the DCG. While the host activates immune signaling to fight infection, intestinal epithelial cells react to aerobic glycolysis by quickly transferring bioenergetic molecules, which causes oxygenation of the epithelium, an instant increase in mucosal-associated commensal Enterobacteriaceae and a decrease in obligate anaerobes (51).

In the case of gut microbial taxa in balanced-constitution people, *Bacteroides plebeius*, *Slackia piriformis*, *Clostridium*_sp_L2_50 and *Veillonella* unclassified were found notably based on LEfSe analysis. The abundance of *Bacteroides plebeius* was mostly attributed to MCH, LYM, TG and WBC counts. It was positively associated with Hb and RBC counts, negative associated with the tendency for anemia mediated by L-Glutamine and D-Glutamine, negative associated with MCHC mediated by alpha−Santalyl phenylacetate in this study, suggesting that *Bacteroides plebeius* might help avoid anemia and might be related to immunity. *Bacteroides plebeius* is related to the dietary components of seaweed and coffee phenolic compounds (52, 53). Other research has suggested that intestinal microbes support the human body in energy from dietary polysaccharides via carbohydrate active enzymes, which are lacking in the human genome, possibly from seaweed-associated marine bacteria (54). One report has shown that, compared to Crohn’s disease patients with recurrence after surgery, remitted patients had higher proportions of *Bacteroides plebeius*, which is a relative of butyrate-producing bacteria (55). Although *Slackia piriformis* and *Clostridium_*sp_L2_50 were rarely reported previously, we found a positive association between *Slackia piriformis* and TBA (*p* < 0.01) and a positive association between *Clostridium*_sp_L2_50 and IL27 (*p* < 0.05), implying that these bacteria are probably probiotic. Based on SPEC-OCCU analysis, *Parabacteroides goldsteinii*, as a key bacterial marker of BCG, is a SCFA producer and possesses gut barrier-maintaining functions and potently protects against pathogenic bacterial lipopolysaccharide-induced inflammation (56). The puzzle appeared when *Clostridium leptum* was increased significantly in the BCAG in the comparison among the four groups, which is acknowledged as a probiotic. *Clostridium leptum* is a member of the Ruminococcaceae family acknowledged to be capable of 7α-dehydroxylation and producing secondary BAs that mainly have anti-inflammatory activities (57, 58). The SCFAs generated by this community modulate the expression of Foxp3, an important gene governing the development of regulatory T cells (59). This may indicate that although people were out of the normal blood index range and tended to have anemia, they may still take advantage of their balanced constitution.

We then went a step further to reveal that people of different constitutions tend to have anemia or avoid anemia under the influence of their particular intestinal microbiota. Distinguished from people of balanced constitution, the differential microbiota of people with deficient constitution showed less auspicious results, although they were all within the normal blood index ranges at the time. In addition to *Clostridium bolteae*, *Streptococcus parasanguinis* and *Flavonifractor plautii*, distinguished as characteristic microbiota in the DCG as stated, *Bacteroides thetaiotaomicron*, *Streptococcus australis*, *Streptococcus salivarius* and *Lachnospiraceae* bacterium_7_1_58FFA showed markedly higher abundance in the DCNG. *Streptococcus australis*, *Streptococcus parasanguinis* and *Streptococcus salivarius*, as oral bacteria, are usually found along with each other, and they are related to precancerous lesions of gastric cancer, Takayasu arteritis, COPD and COVID-19-related mortality (60–63). On the other hand, *Bacteroides thetaiotaomicron* has a strong ability to degrade dietary polysaccharides, produce host-absorbable short-chain and organic acids as a resource of energy, transform cholesterol to cholesterol sulfate, and ameliorate inflammation by producing bacterial extracellular vesicles (64–66).

However, when we focused on people out of the normal red blood cell index ranges, the differential microbiota of people with a balanced constitution showed good indications compared to people with a deficient constitution. *Akkermansia muciniphila, Eubacterium eligers, Alistipes finegens, Bacteroides nordii, Bacteroides plebeius, Parabacteroides goldsteinii* and *Clostridium leptum* were enriched in the BCAG, most of which are regarded as probiotics. These results demonstrate that people with tendency for anemia but a balanced constitution may be in a more beneficial situation than those with a deficient constitution. *Akkermansia muciniphila*, *Eubacterium eligers*, and *Parabacteroides goldsteinii* are all SCFA producers (59, 67). *Akkermansia muciniphila* can maintain gut barrier function, reorganize disturbed microorganisms, enhance SCFA secretion, and alleviate APAP-induced oxidative stress and the inflammatory response (68, 69). *Parabacteroides goldsteinii* also possesses gut barrier-maintaining functions and potently protects against pathogenic bacterial lipopolysaccharide-induced inflammation (56). For the characteristic gut microbiota in the DCAG, *Bacteroides vulgatus* and *Ruminococcus gnavus* were considered hominoxious and reported to be related to inflammatory bowel disease, metabolic syndrome, postacute COVID-19 syndrome, spondyloarthritis and type 2 diabetes mellitus and other diseases (70–75). Moreover, *Ruminococcus gnavus* was a key bacterial marker in the DCG, with higher occupancy and specificity in deficient-over balanced-constitution samples in this study. We consider *Ruminococcus gnavus* to be a shared microbial basis of deficient constitution and tendency for anemia.

According to previous research, anemia is related to alterations in the gut microbiome (26–30). In our study, aerobic and facultative-anaerobic bacteria were more abundant in the DCG, while its anaerobic bacteria were less abundant. As we known, low availability of oxygen characterizes the intestinal lumen. The consumption of oxygen by mammalian intestinal epithelial cells (IECs) leads to intestinal hypoxia, which is maintained by SCFAs produced by some specific microbiota (especially by anaerobic bacteria). This finding provides evidence that the change of intestinal anaerobic environment may be associated with deficient constitution.

Metabonomics provides some clues for identifying differences between deficient constitution and balanced constitution individuals. In brief, samples with a deficient constitution were more likely to have energy metabolism disorders, including amino acid metabolism and glycolipid metabolism, associated with low-energy and immunologic derangement. 2-Oxoadipate (upregulated metabolite) can be converted to acetyl-CoA in mitochondria (76), and abnormal quantities of 2-aminoadipic acid are found in patients with 2-oxoadipic aciduria (77). Cucurbitacin C (downregulated metabolite), with antioxidant ability shows growth inhibition capabilities against tumor cells by activating cellular immunity (78). Cucurbitacin D promotes fetal hemoglobin synthesis by activating the p38 pathway and stabilizing γ-globin mRNA (79). Decanoylcarnitine (downregulated metabolite), an acylcarnitines, can produce energy by transporting and breaking down organic acids and fatty acids (80). Tauroursodeoxycholic acid (downregulated metabolite), a type of bile acid, is an essential component for cholesterol homeostasis, by regulating the secretion of bile and lipids for the digestion of dietary fats and vitamins (81). A previous study in mice reported that the LPS-induced inflammatory damage group had lower levels of glycerol triundecanoate (downregulated metabolite) than the control group (82).

After adjusting fo age, BMI, MCHC, MCH, MCV, RDW, HCT, RBC count and Hb level by covariate adjustment analysis, L-gluconolactone, pentanenitrile, gluconolactone and hydroxyoctanoic acid were significantly more abundant in the DCG. Gluconolactone is generated by enzymatic oxidation of D-glucose by the enzyme glucose oxidase, with antioxidant free radical scavenging activities. Additionally, the most significant biological pathway in the DCG was related to the pentose phosphate pathway, which is the first-line defense response to oxidative stress. In addition to being absorbed from the diet, gluconolactone is one of the metabolites of the gut microbiota, affecting biological and pathological processes once intestinal homeostasis changes (83). Gluconolactone in feces increased after a half-marathon race and after a high-fat diet (84, 85), indicating that gluconolactone may play an important role in the gut microbiota and human health. L-Gulonolactone and pentanenitrile come mostly from a few particular food and have uncertain roles in humans.

Lower Hb counts was observed in the DCG, and galacturonic acid displayed a negative linear relationship with Hb in this study. Hb is formed by α- and β-globin subunits attached to heme prosthetic groups, which can transport oxygen and carbon dioxide. Hb molecules limit possible pathologies caused by associated iron and free oxygen, reactive molecules capable of inflicting damage through the production of reactive oxygen species (86). It is believed that modification of HbA at its amino terminus with galacturonic acid influences the O_2_ affinity of the molecule (87). The top 3 characteristic bacterial constituents of the deficient constitution, the Gammaproteobacteria class, Bacilli class and Lactobacillales order, contributed to the enrichment of metabolic pathways of galacturonic acid. Pectin oligosaccharides, emerging prebiotics consisting of both galacturonic acids and neutral sugars, have an effect on the microbiome, and the latter also impact the cholesterol-reducing effects of pectin oligosaccharides via specific bacteria and their SCFA metabolites (88). Therefore, we assumed that the microbiome of the DCG may also impact the Hb-regulating effects of galacturonic acids, but the specific mechanisms need further study.

Metabonomics analysis was applied to distinguish between the AG and NG. Generally, individuals with a tendency for anemia were in the DCG and mainly had deficient energy metabolism and immunologic derangement. Recently, 2-hydroxybutyrate (upregulated metabolite) in the plasma has been noted as a favorable indicator for type 2 diabetes in the early-stage and is commonly found in the urine of lactic acidosis and ketoacidosis patients, which have high concentrations of it related to energy-deficient metabolism (e.g., birth asphyxia) (89–91). L-Methionine (upregulated metabolite) is a necessary amino acid, but persistently excessive methionine is related to homocystinuria-megaloblastic anemia (92). Cholic acid (downregulated metabolite), as primary bile acid, maintains cholesterol homeostasis by regulating all vital enzymes (93).

The human gut microbiome is fed by dietary nutrition to generate bile acids, SCFAs and other bioactive compounds, which are necessary for maintaining host physiology involving in protein metabolism, glucose metabolism and lipid metabolism (94). Herein, we used multiple technologies including metagenomics and metabolomics, to identify, categorize, and quantify compounds of deficient individuals, and found that decreased bacteria relevant to SCFA production and bile acid secretion, which influence energy metabolism and inflammation, are prominent features of deficient individuals. SCFA production is one of the well-known metabolic attributes of human gut bacteria and is connected with health situations (95). They can boost mitochondrial β-oxidation, deplete oxygen and consequently reduce the degradation of hypoxia induction factor (HIF), affecting host ATP production and immune responses (96–98). HIF-1 can influence the barrier function of the epithelium by regulating the expression of several relevant genes (96). HIF-2α is a master transcription factor of intestinal iron absorption and is related to intestinal iron absorption in the host. It has been suggested that activation of HIF-2α by microbiome-based therapeutics would lead to increased intestinal iron absorption and alleviate anemic disorders (99). In addition, because of lower Hb counts in people with deficient constitution, cells exposed to prolonged hypoxia may activate HIF (100), and metabolic pathways of the defense response to oxidative stress were observed in this study.

This study may have some potential limitations. We used a convenience sample due to the limitation of resources, and it may cause sampling bias because of geographic specificity, diet and other confounding factors. This study is a cross-sectional study, the causal inference and the identified associations need further studies.

To our knowledge, this study comprehensively investigated a relatively unexplored area—the relationship between alterations in the gut microbiome due to deficiency constitution and the tendency toward anemia. In the future research, larger sample sizes and longitudinal cohort studies will be performed to reveal the more specific associations between them and offer potential interventions to reduce the risk of anemia in individuals with deficiency constitution.

## 5 Conclusion

Our study suggests that the composition of the gut microbiome differs between deficient-constitution and balanced-constitution subjects. Alterations in the microbiome of people with deficient constitution were associated with worse health status and a greater risk of anemia, involving intestinal barrier function, metabolism and immune responses, which were regulated by SCFAs and bile acid production. These findings may offer a new perspective of traditional Chinese medicine constitution regarding the etiology of anemia with implications for developing new strategies for prevention of anemia.

## Data Availability

The datasets presented in this study can be found in online repositories. The names of the repository/repositories and accession number(s) can be found at: https://www.ncbi.nlm.nih.gov/genbank/, CNP0003844.

## Footnotes

^1^ www.metlin.scripps.edu

^2^ www.hmdb.ca

^3^ www.genome.jp/kegg

^4^ http://www.metaboanalyst.ca/

^5^ http://metorigin.met-bioinformatics.cn
