# Supplementary material for: The increased tendency for anemia in traditional Chinese medicine deficient body constitution is associated with the gut microbiome
Source: Front Nutr. 2024 Sep 18;11:1359644. doi: 10.3389/fnut.2024.1359644 (PMC11445043; doi:10.3389/fnut.2024.1359644)
Supplement: Supplementary file 2 [file Data_Sheet_1.docx]

**Appendix A. Supplementary data**

A


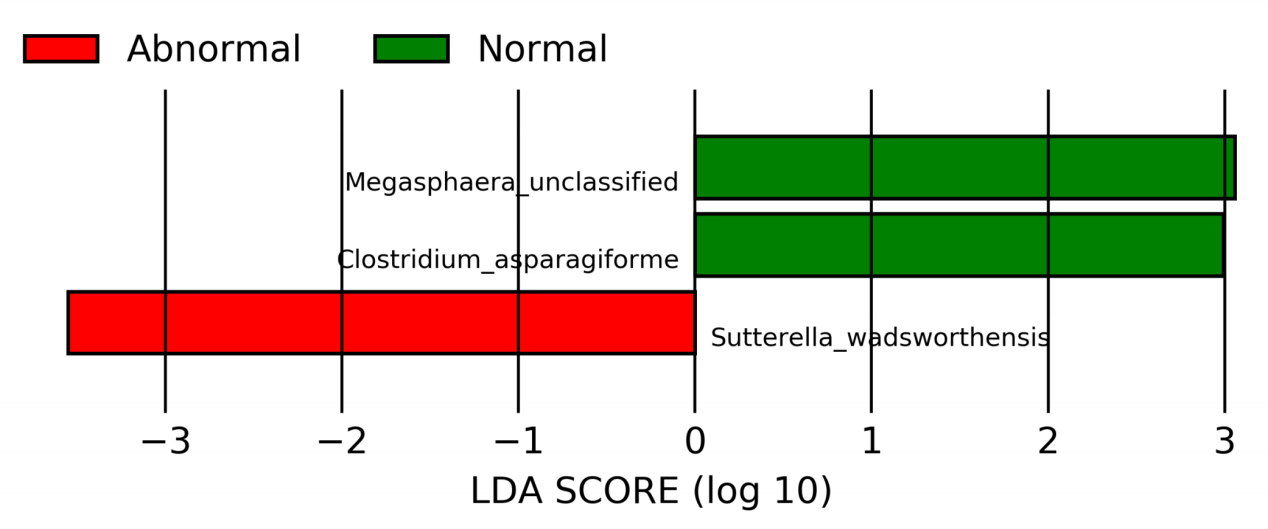


B


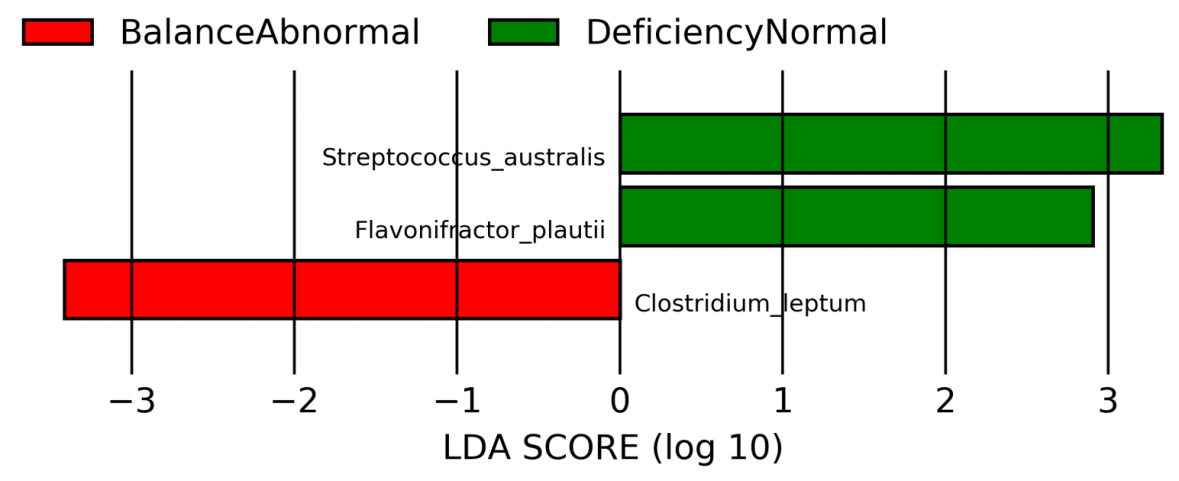


C


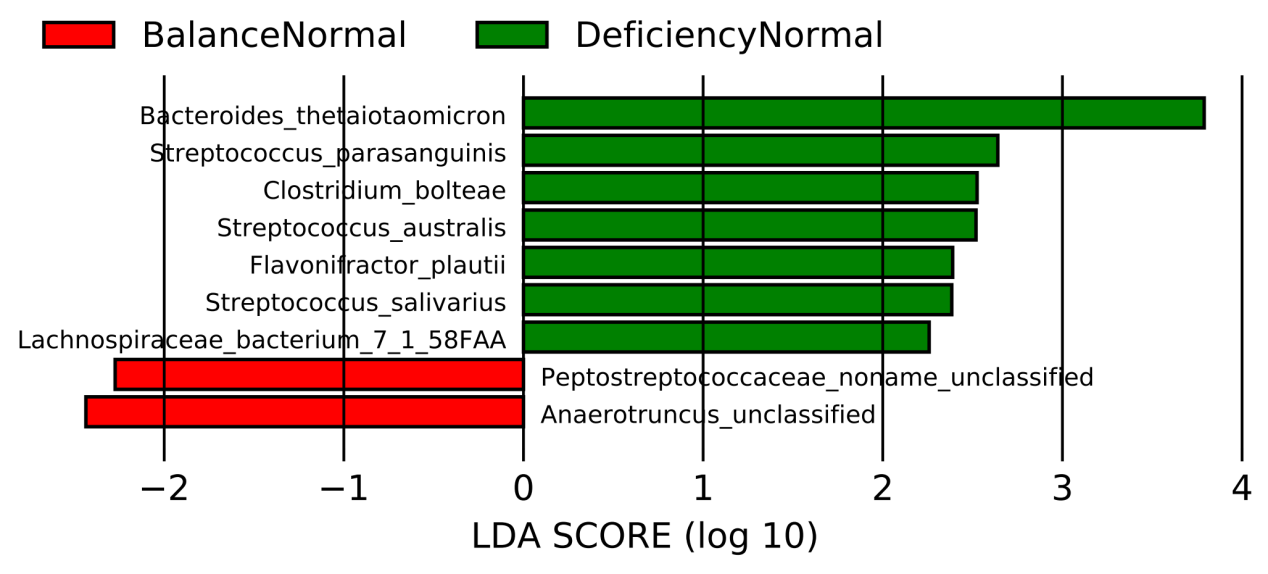


D
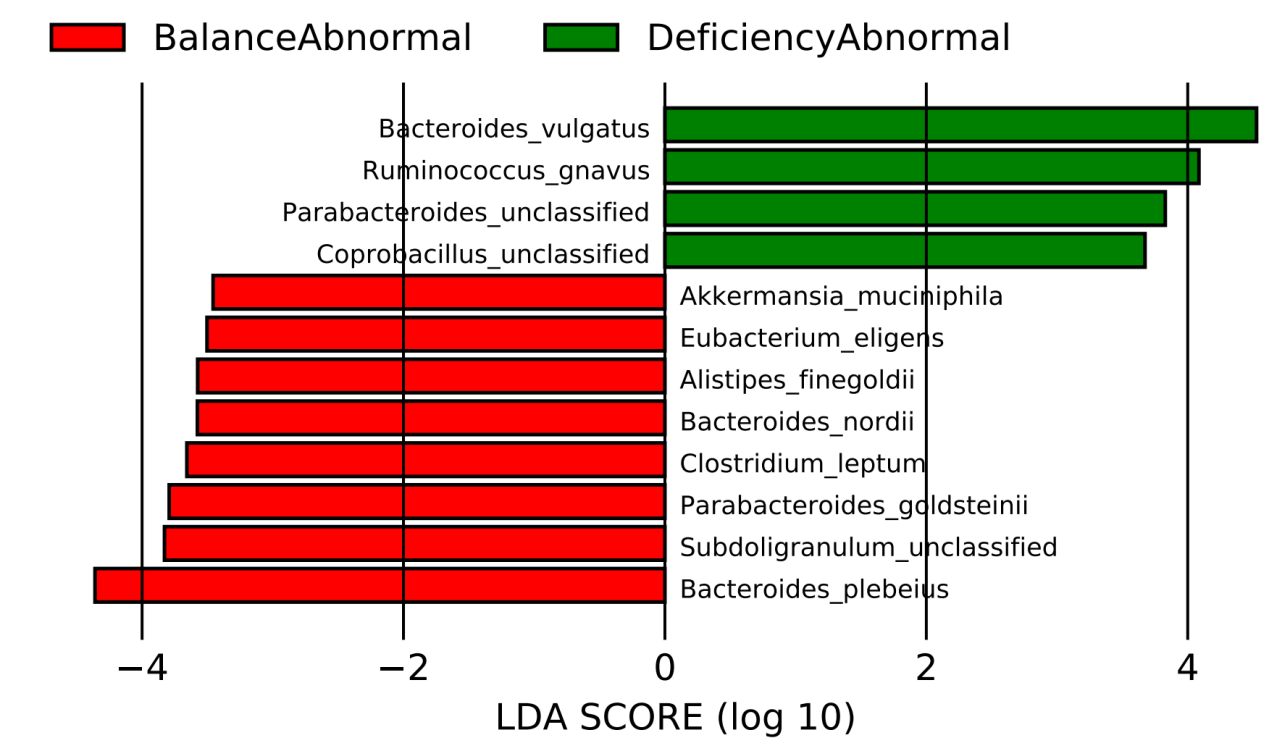


Fig. S1. Histogram of LDA logarithmic scores of biomarkers found by LEfSe (P< 0.05 and the LDA cutoff >2.0) for the species level features differentially abundant (A) between AG and NG, (B) among DCNG, DCAG, BCNG and BCAG, (C) between DCNG and BCNG, (D) between DCAG and BCAG.


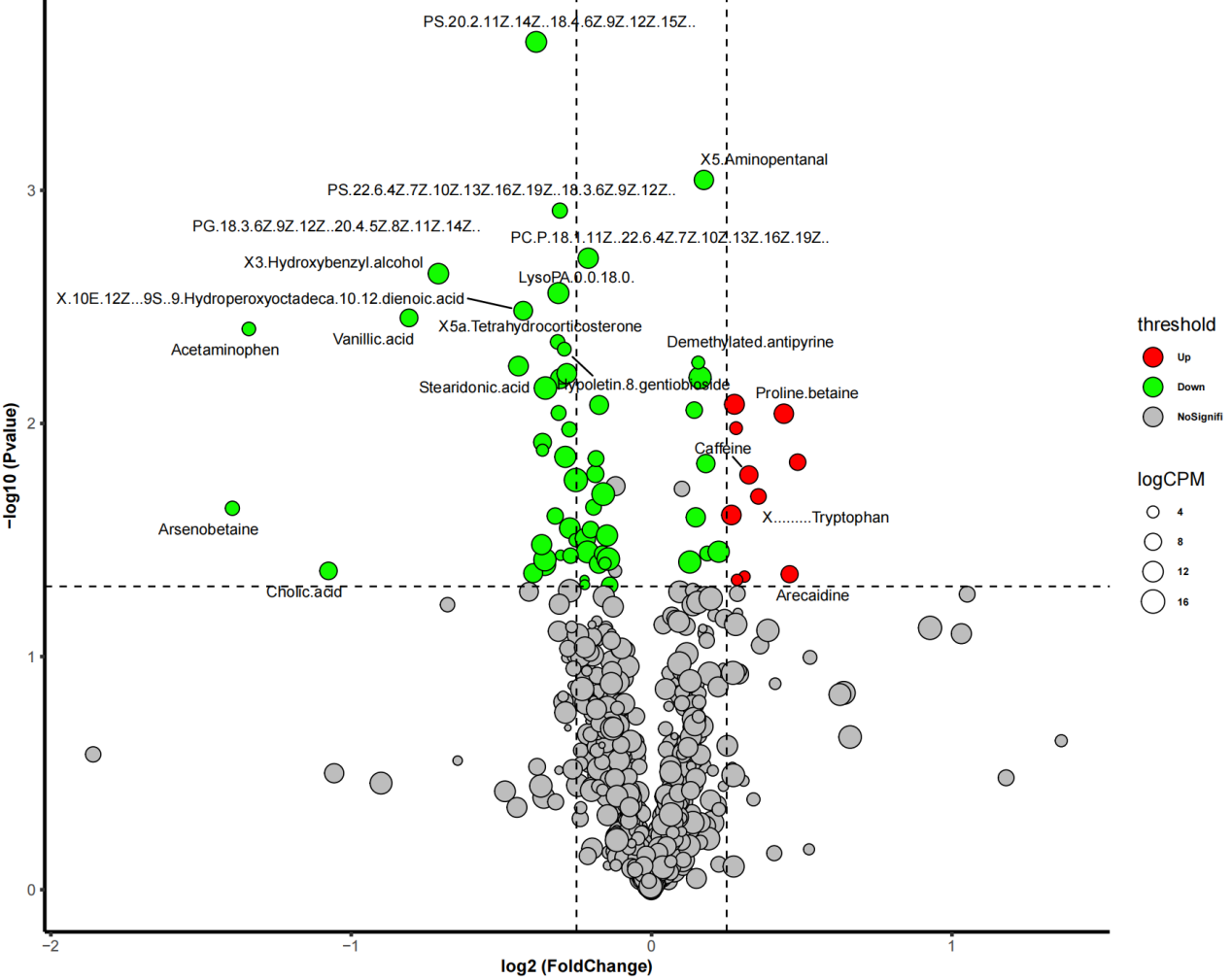


Fig. S2. Volcano plot of the differential metabolites between AG and NG.


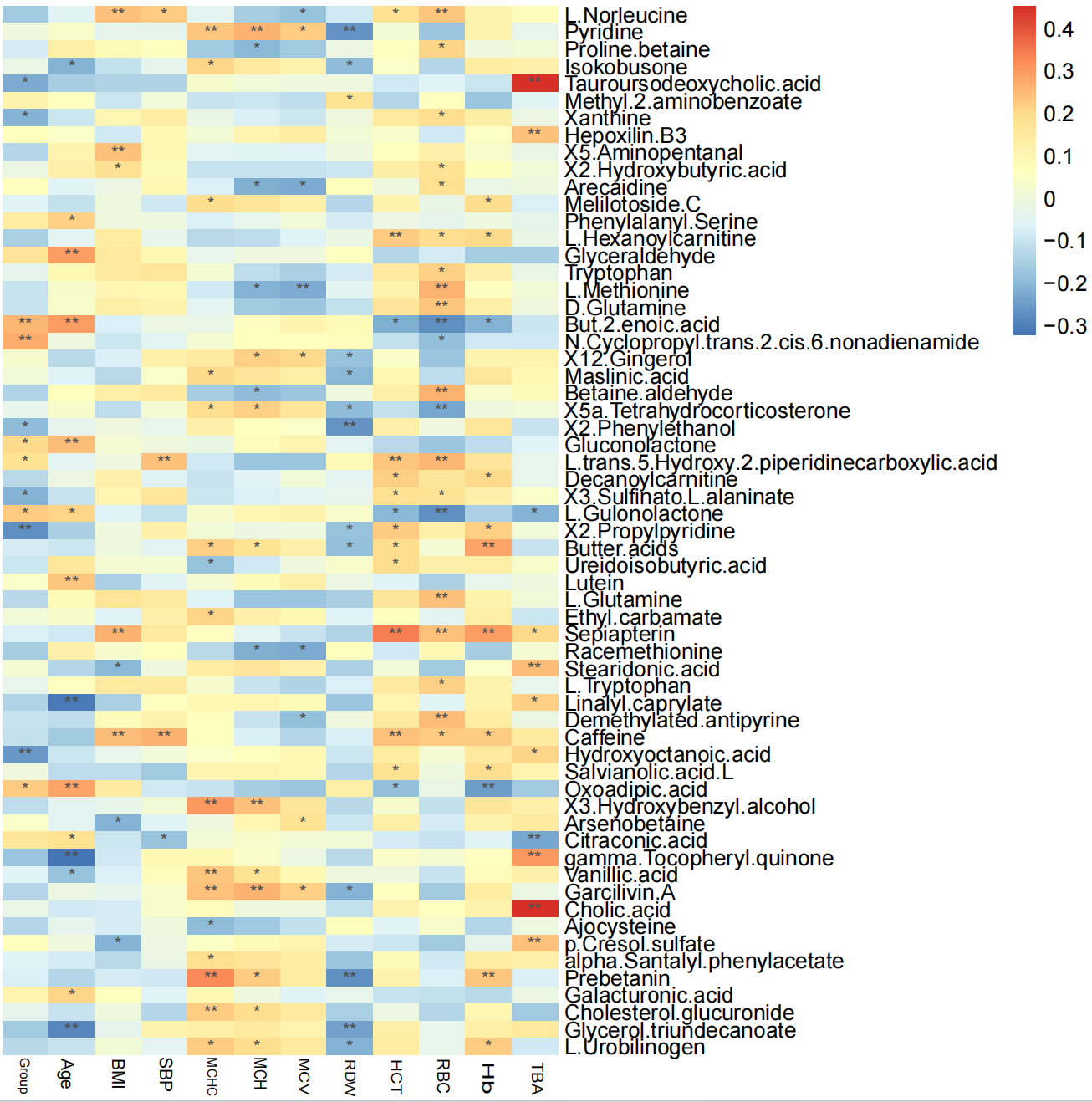


Fig. S3. Heatmap based on Spearman's correlation coefficients to measure the linear relationship between clinical index and metabolomic features. X-axis: clinical index; Right y-axis: metabolites. Color depth indicates the level of positive (red) or negative (blue) correlation, and asterisks indicate significance (**P* < 0.05, ***P* < 0 .01).


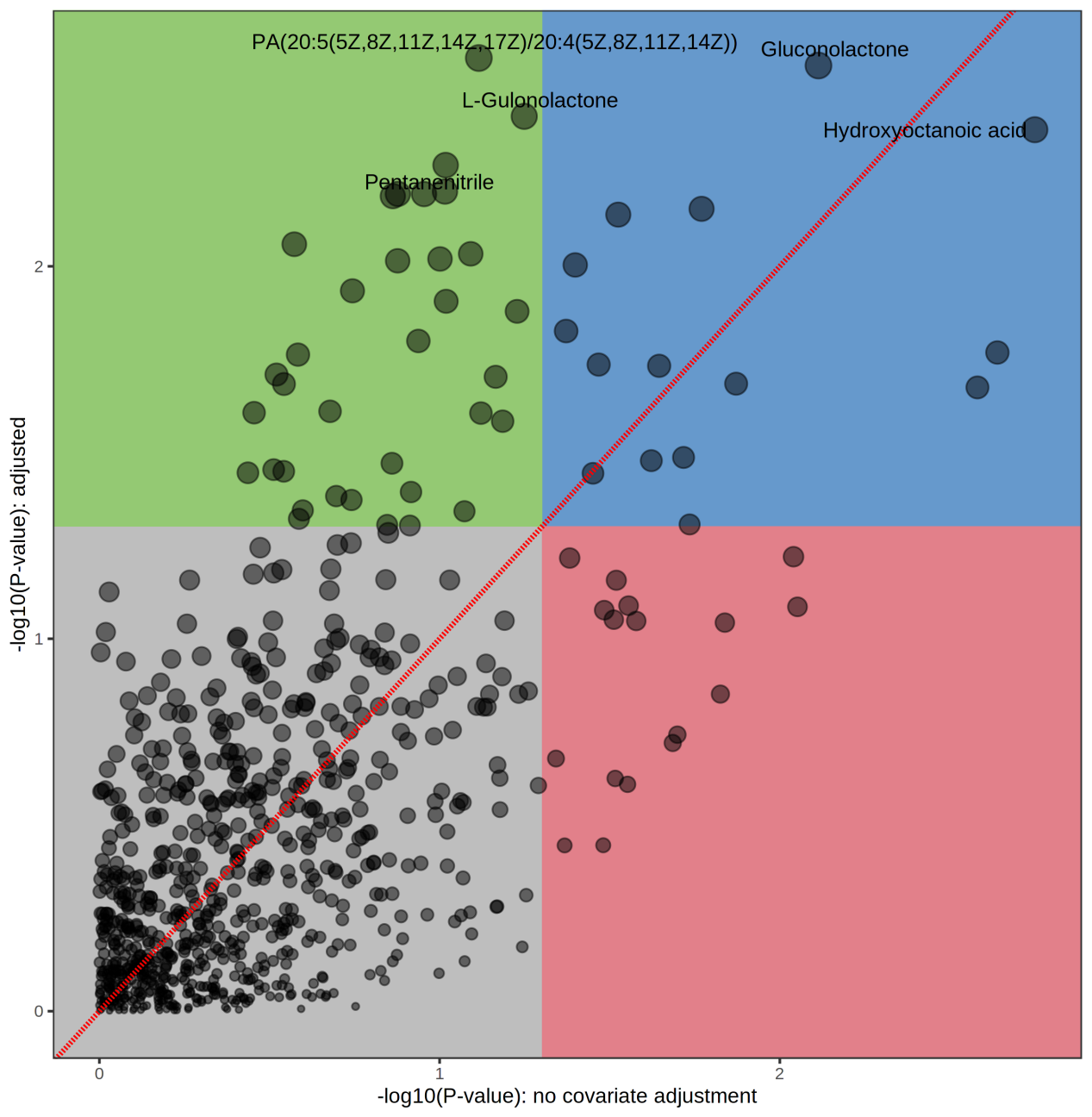


Fig. S4. Linear models with covariate adjustment analysis of metabolites in the DCG adjusting differential clinical features including age, BMI, MCHC, MCH, MCV, RDW, HCT, RBC, Hb counts between the DCG and BCG. The green section shows features significant only after adjustment, red is significant only before adjustment, and blue is significant in both cases.

A B


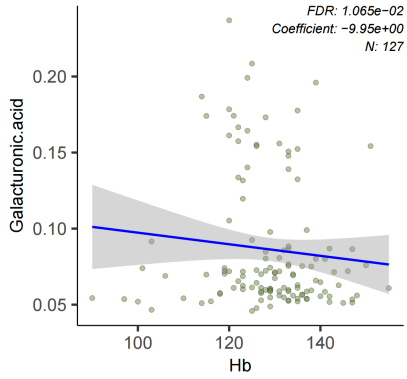

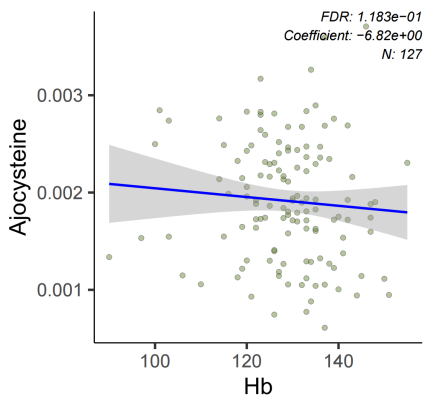


C


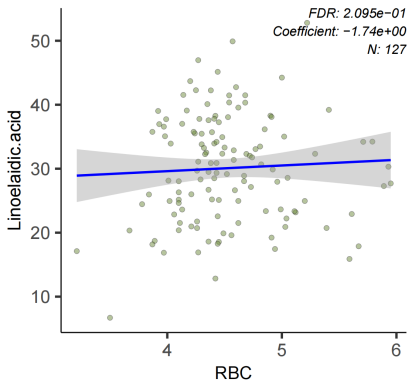


Fig. S5. Based on Maaslin2 analysis to determine multivariate associations between clinical data and metabolomic features in the DCG, taking deficiency constitution, age, BMI, MCHC, MCH, MCV, RDW, HCT, RBC and Hb counts into account. (A) Galacturonic acid displayed negative linear correlation with Hb (FDR＜0.001); (B) Ajocysteine displayed negative linear correlation with Hb (FDR＜0.001); (C) Linoelaidic acid exhibited positive linear correlation with RBC (FDR＜0.001).


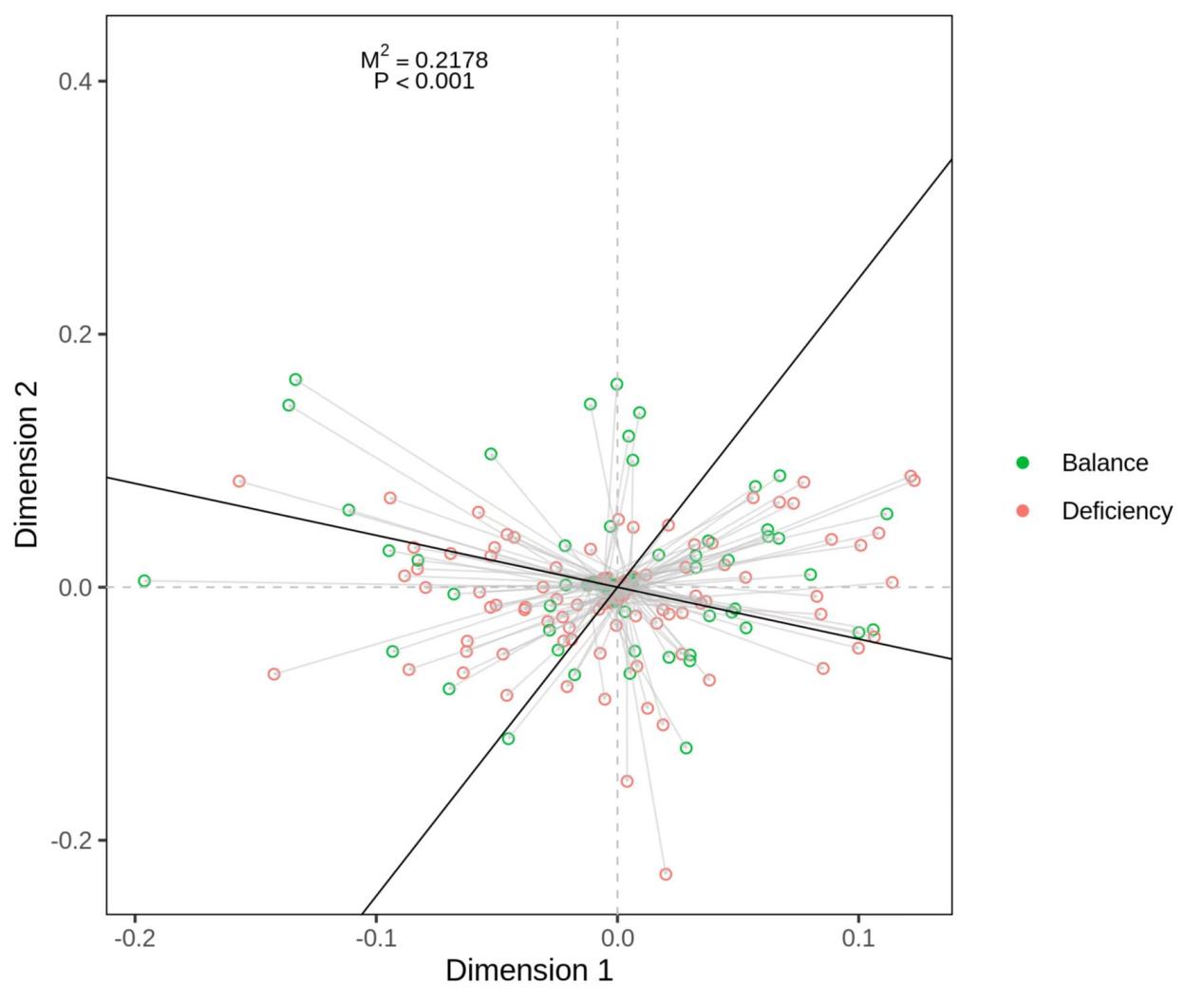


Fig. S6. Procrustes analysis of the correlation between metabolite and microbial community based on the their abundances (M2=0.217, *P*＜0.001).

A


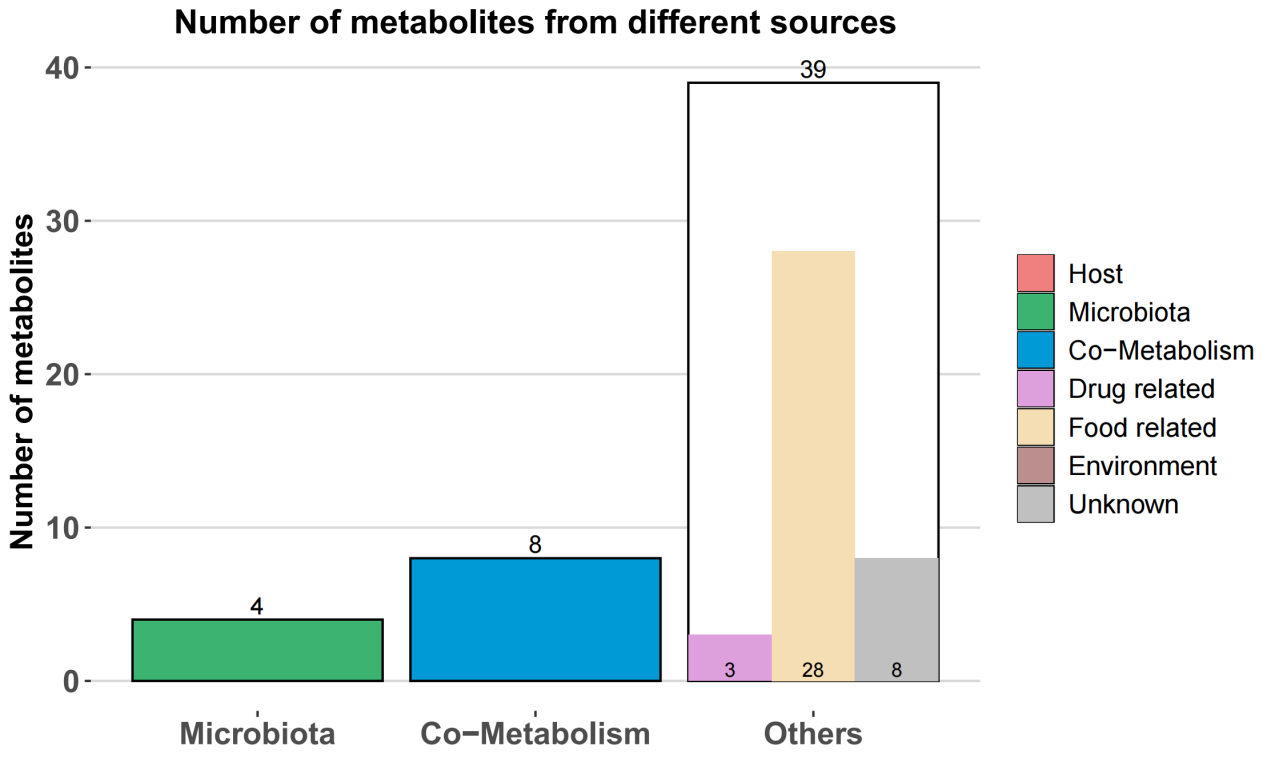


B


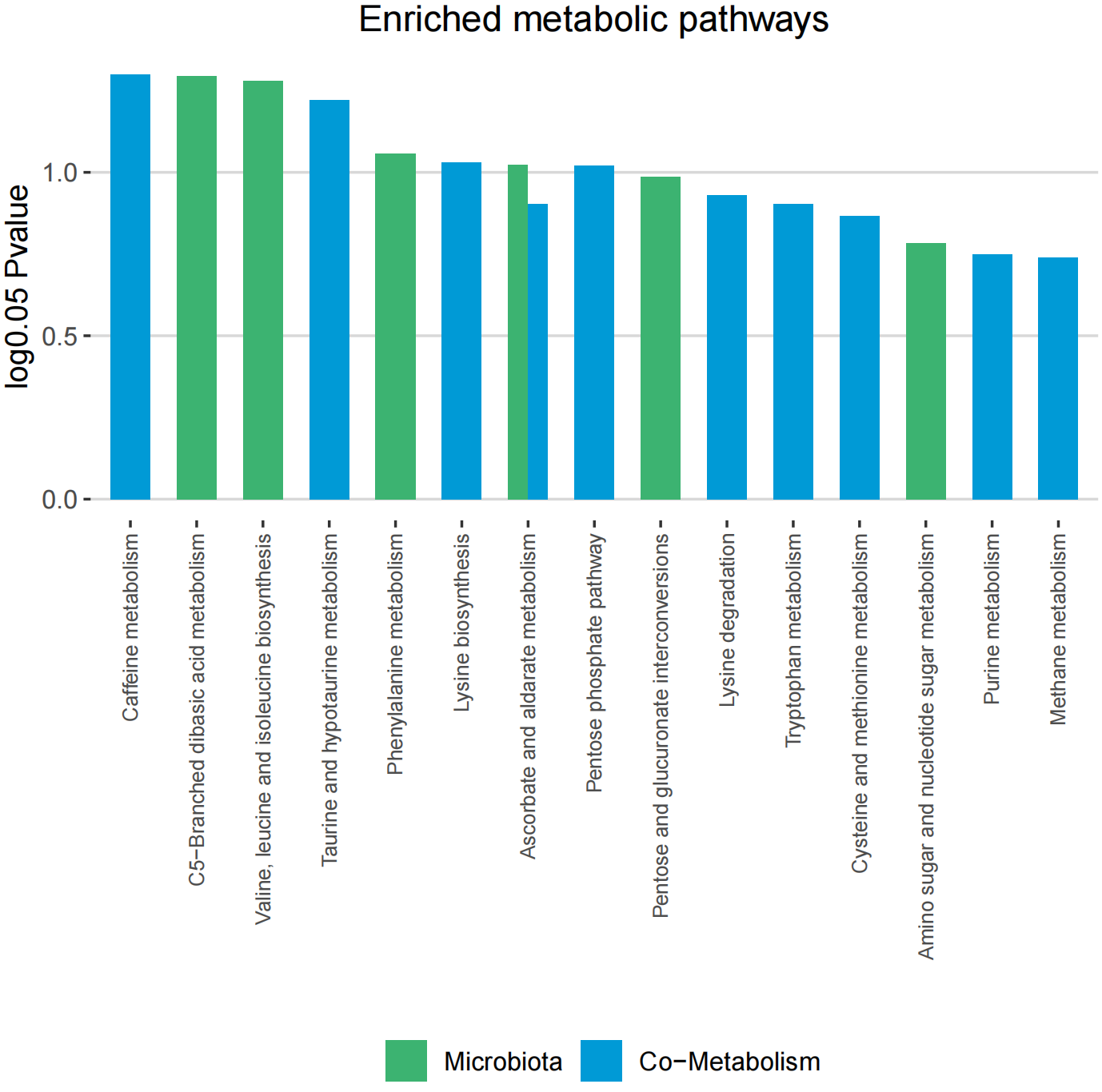


C


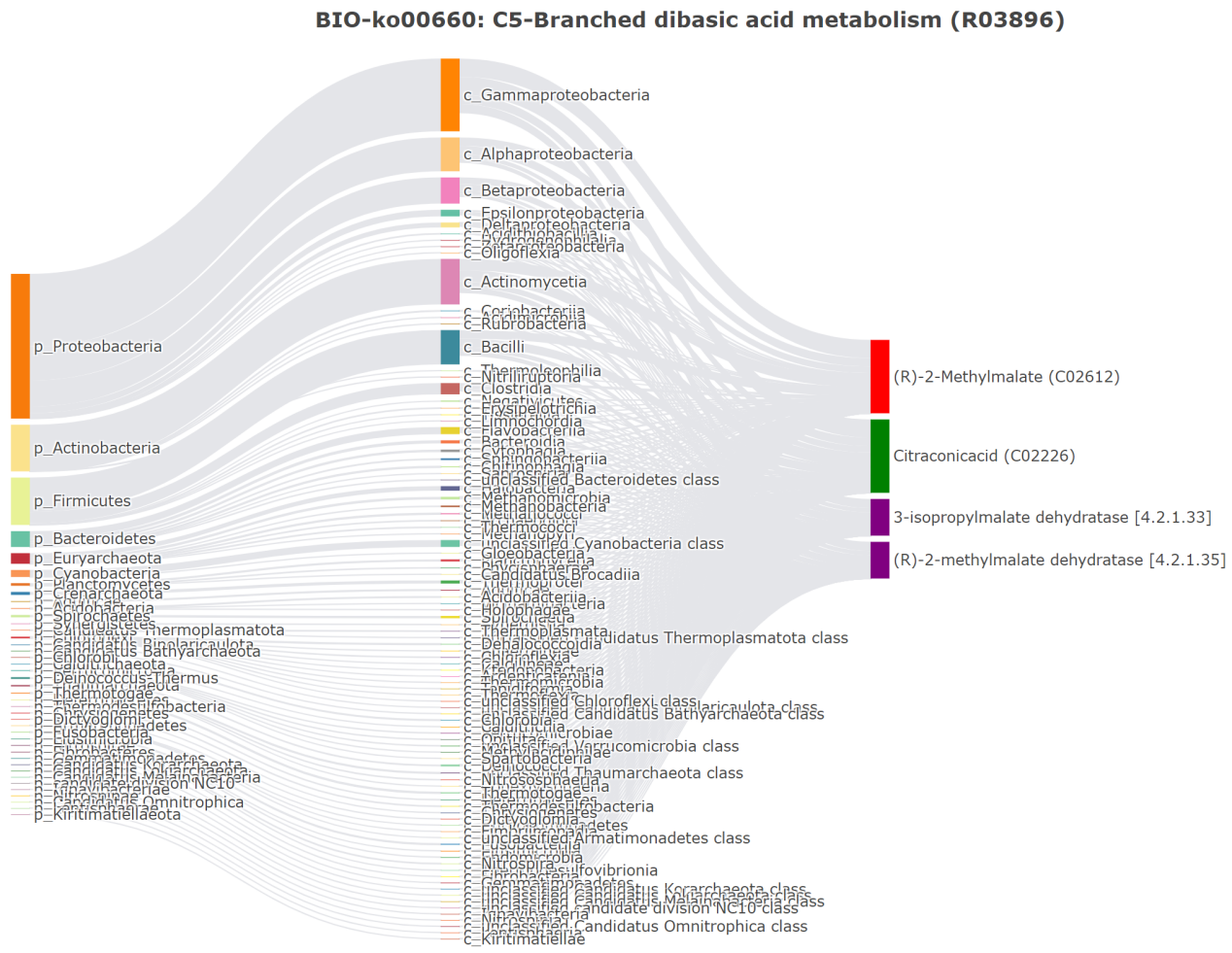


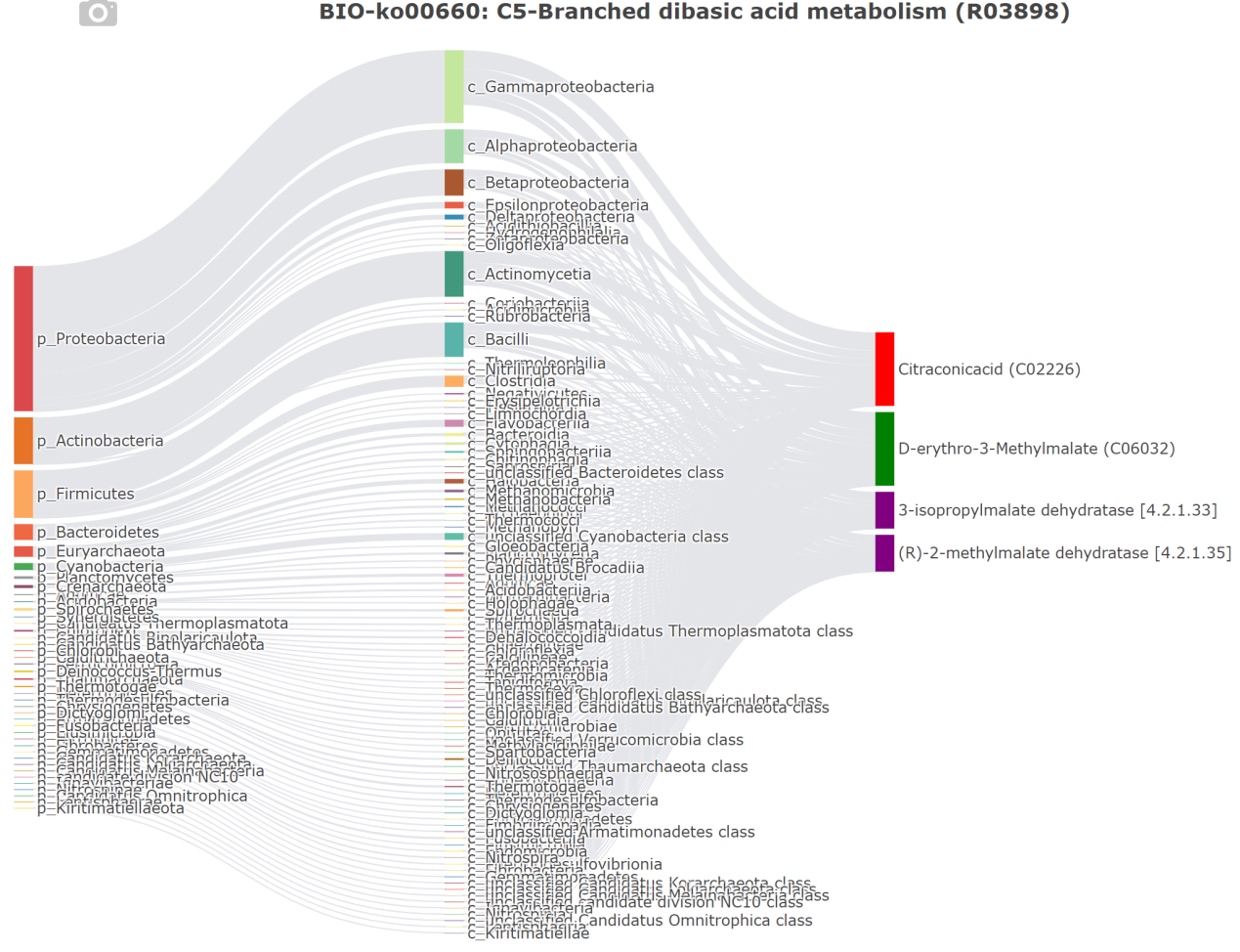


D


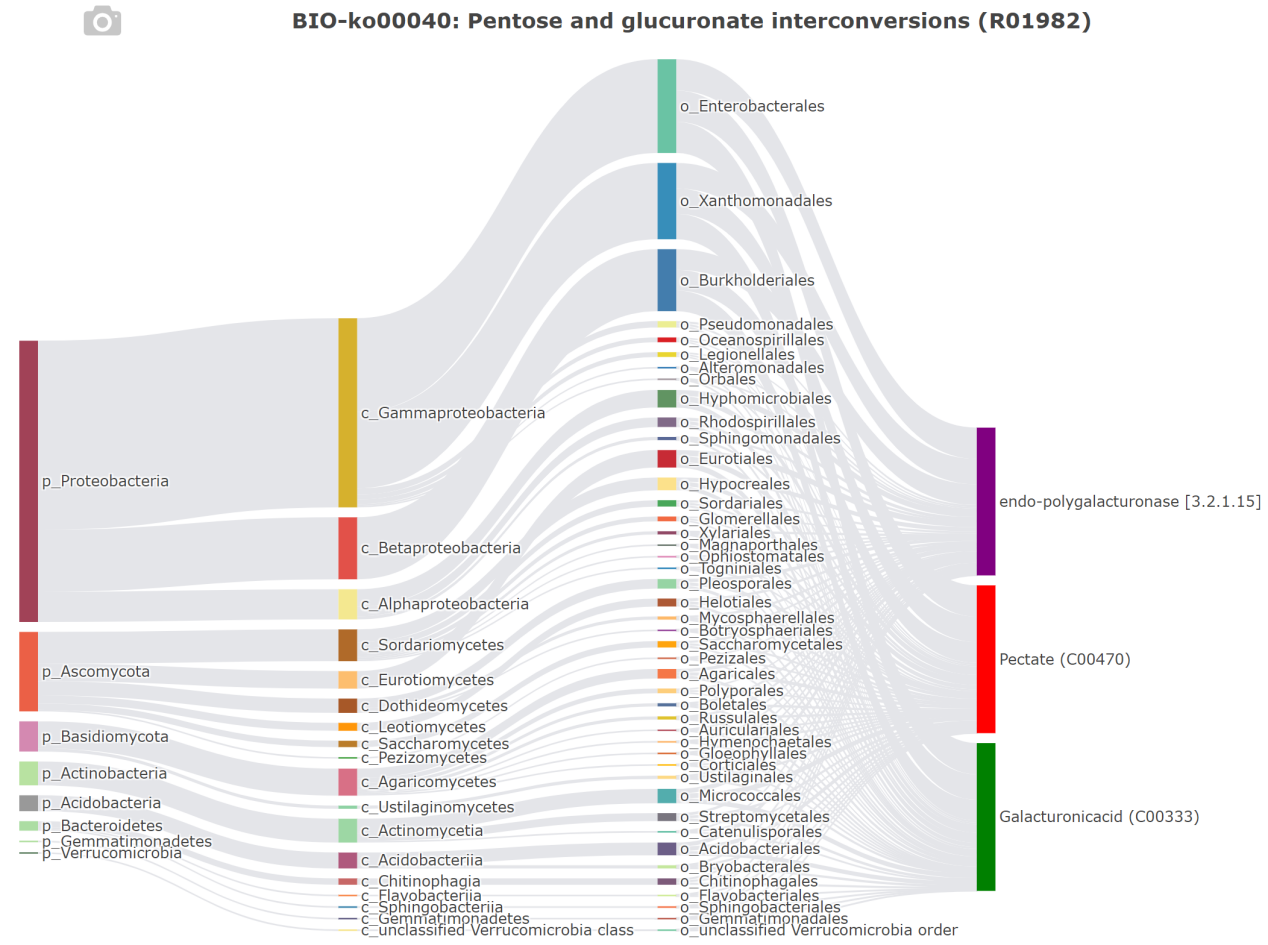


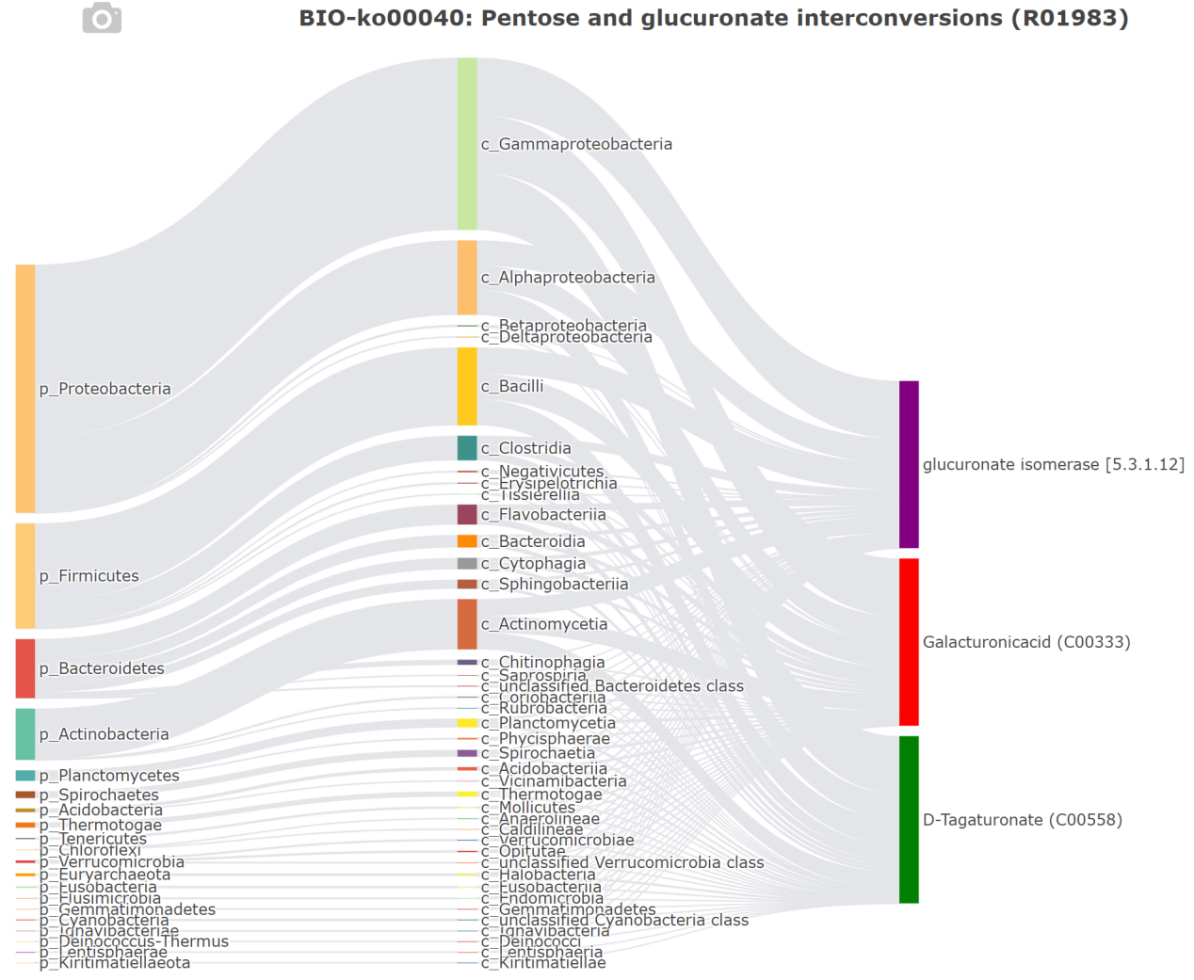


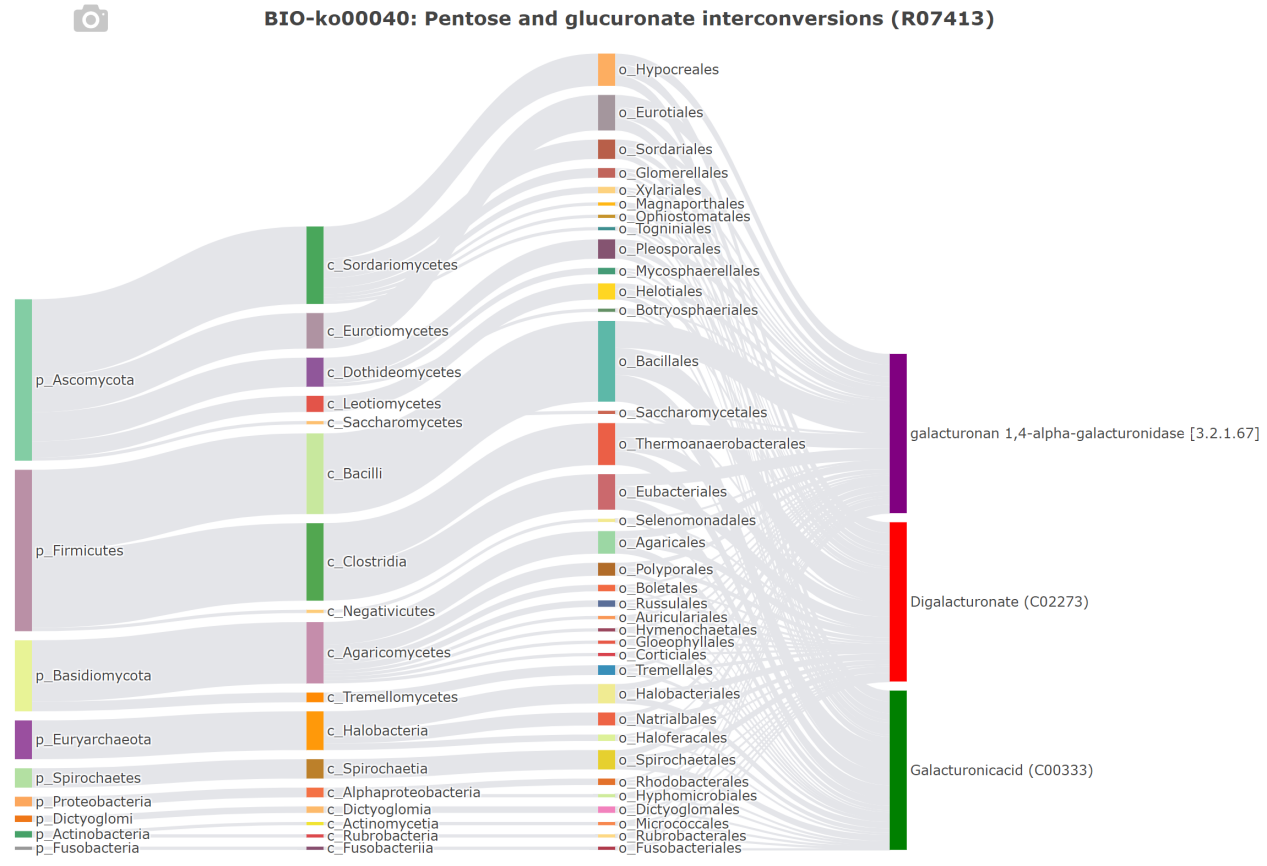

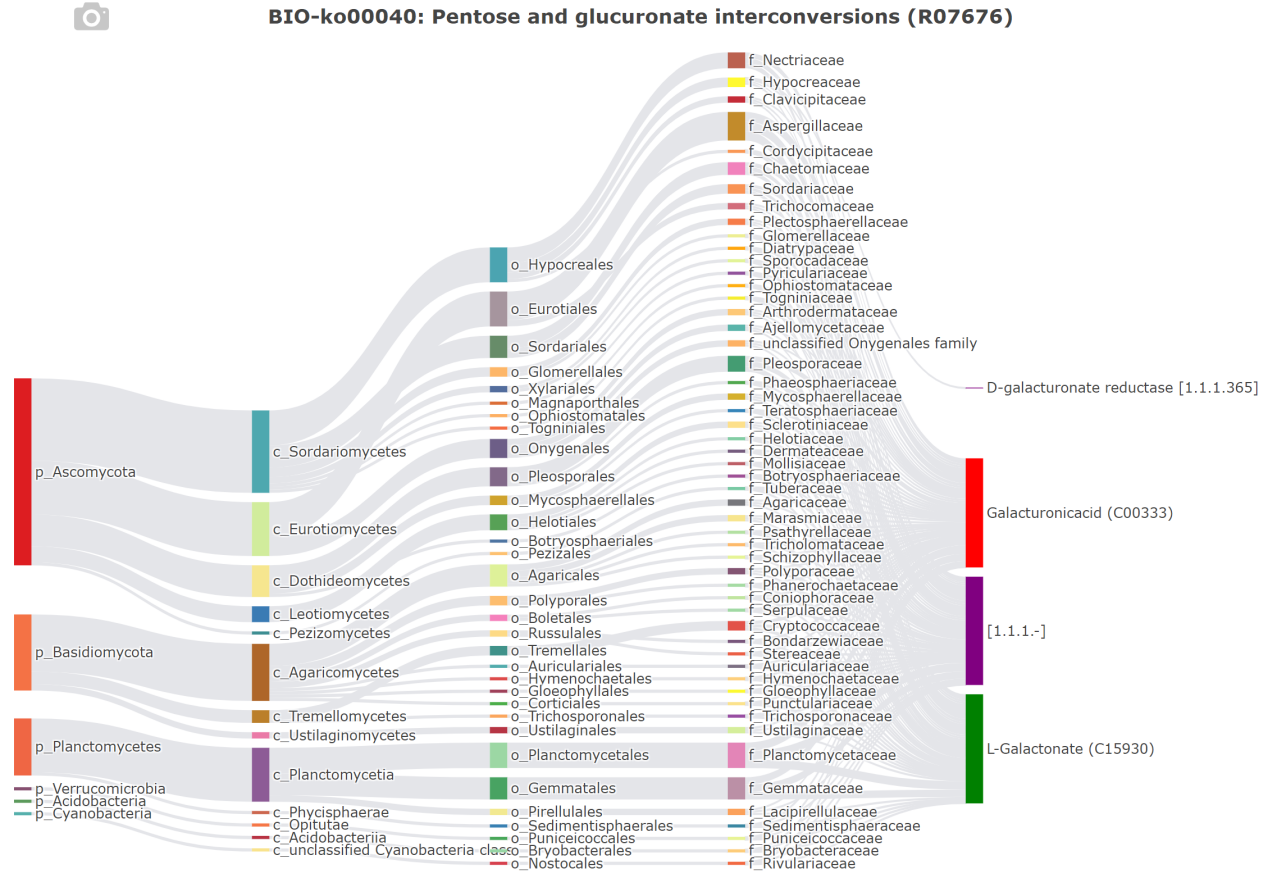

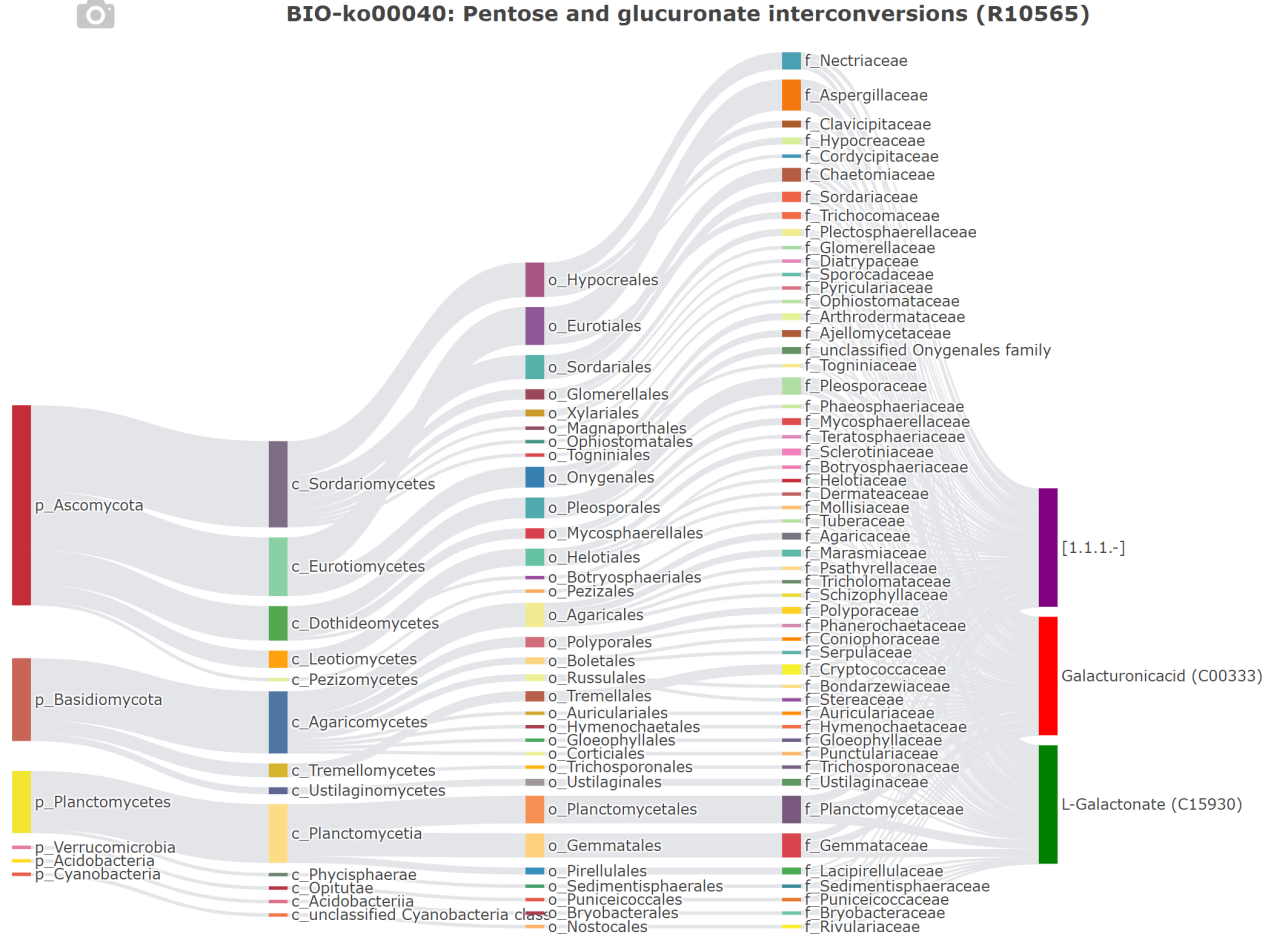


E


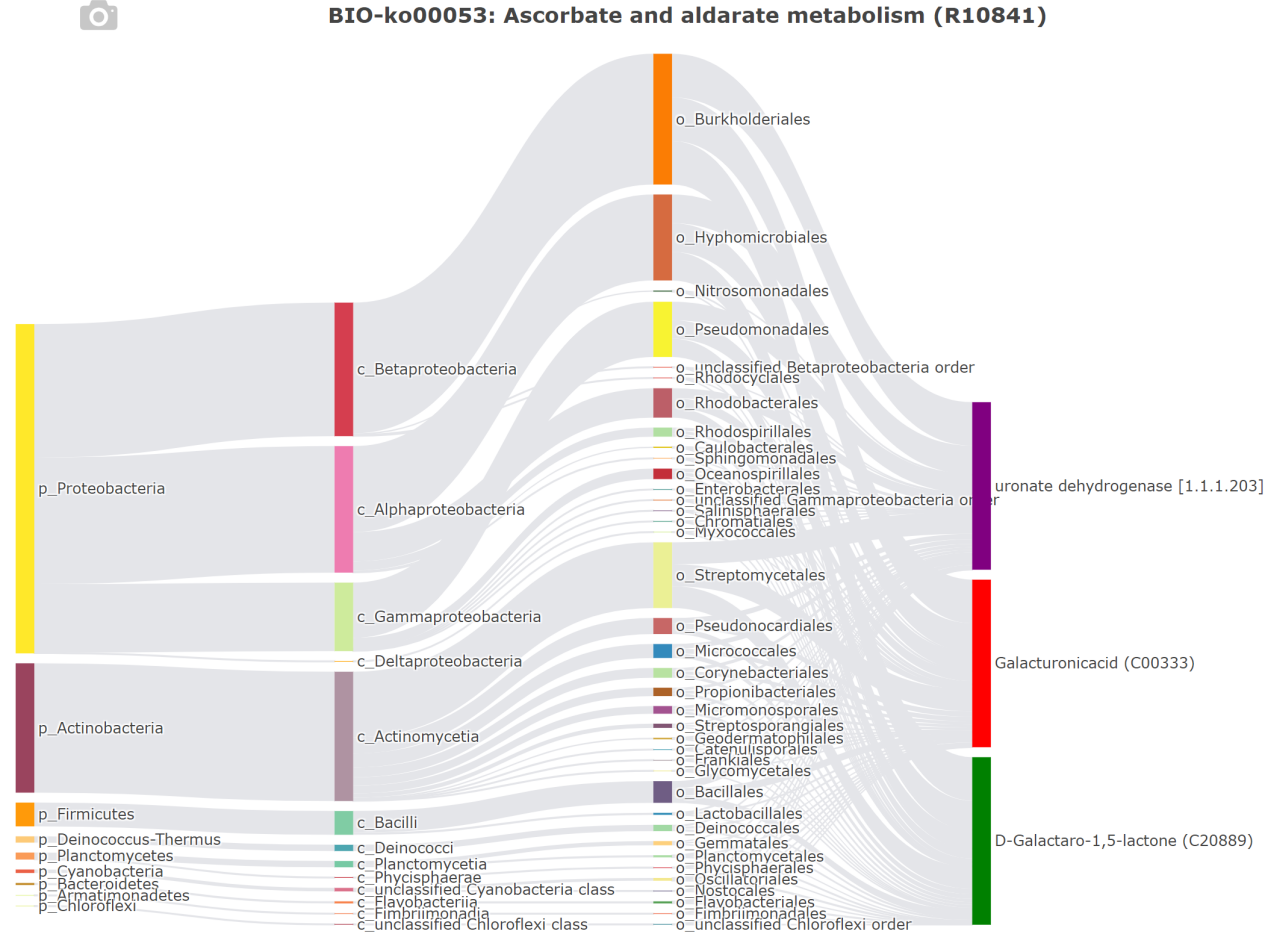


F


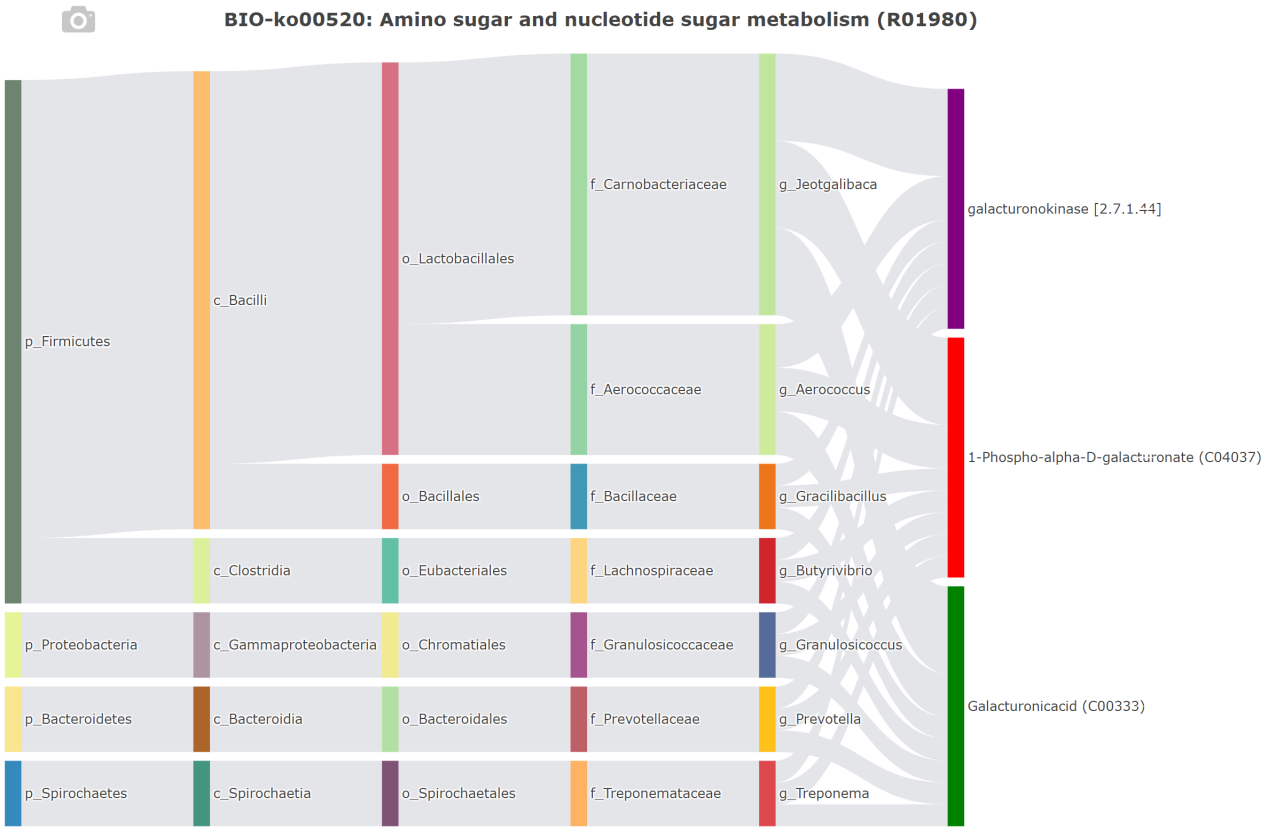


Fig. S7. The complex interplay between microbiome and characteristic metabolites of deficiency constitution and those correlated with Hb and RBC based on MetOrigin. (A) Number of metabolites from different sources. (B) Enriched metabolic pathways. Statistical correlations and biological relationships in the pathway of metabolites: (C) “C5-Branched dibasic acid metabolism”, (D) “Pentose and glucuronate interconversions”, (E) “Ascorbate and aldarate metabolism” and (F) “Amino sugar and nucleotide sugar metabolism”.

A


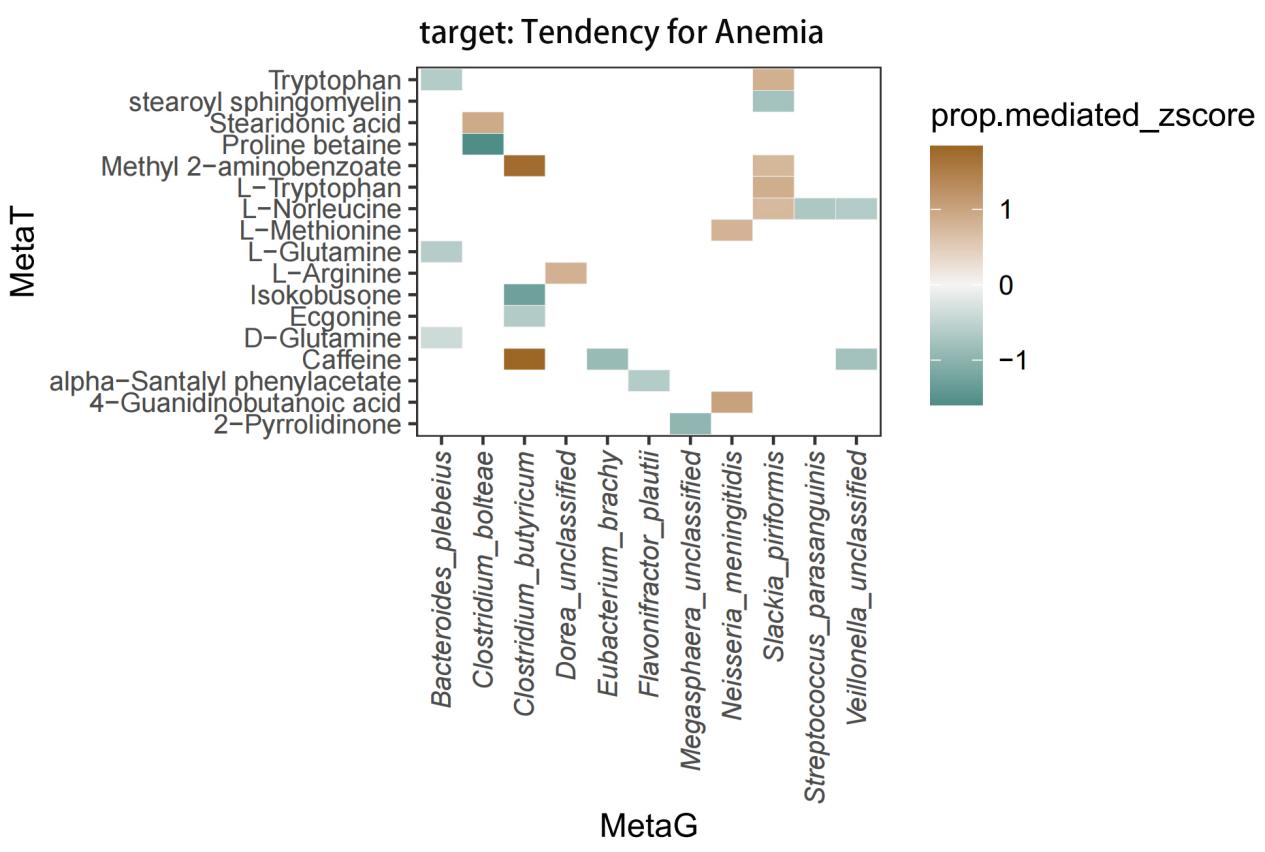


B


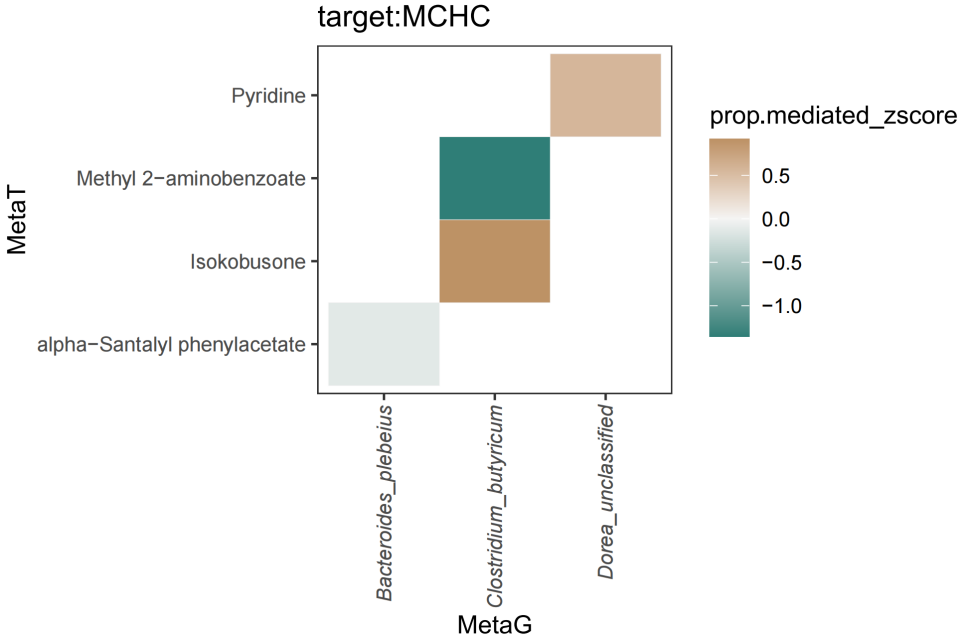


Fig. S8. Full clustered heatmap shows significantly associated metagenome (MetaG) and metabolome (MetaT) features in a sequential mediation analysis for (A)Tendency for Anemia or (B)MCHC. The paired modules with significant mediation effect are colored by their directionality of correlation (*P*<0.05), and those without significant mediation effects are in white.
